# Sensitive and affordable diagnostic assay for the quantitative detection of anaplastic lymphoma kinase (*ALK*) alterations in patients with non-small cell lung cancer

**DOI:** 10.18632/oncotarget.9471

**Published:** 2016-05-19

**Authors:** Elisa Dama, Micol Tillhon, Giovanni Bertalot, Francesca de Santis, Flavia Troglio, Simona Pessina, Antonio Passaro, Salvatore Pece, Filippo de Marinis, Patrizia Dell'Orto, Giuseppe Viale, Lorenzo Spaggiari, Pier Paolo Di Fiore, Fabrizio Bianchi, Massimo Barberis, Manuela Vecchi

**Affiliations:** ^1^ Molecular Medicine Program, European Institute of Oncology, Milan, Italy; ^2^ Division of Epidemiology and Biostatistics, European Institute of Oncology, Milan, Italy; ^3^ Department of Pathology, European Institute of Oncology, Milan, Italy; ^4^ Division of Thoracic Oncology, European Institute of Oncology, Milan, Italy; ^5^ DIPO, Department of Hemato-Oncology and Oncology, University of Milan, Milan, Italy; ^6^ Division of Thoracic Surgery, European Institute of Oncology, Milan, Italy; ^7^ IFOM, the FIRC Institute of Molecular Oncology, Milan, Italy; ^8^ Present address: Advanced Cell Diagnostics, Segrate, Milan, Italy; ^9^ Present address: Division of Immunology, Transplantantion and Infectious Disease, Leukocyte Biology Unit, San Raffaele Scientific Institute, Milan, Italy; ^10^ Present address: Institute for Stem-cell Biology, Regenerative Medicine and Innovative Therapies (ISBReMIT), IRCCS Casa Sollievo della Sofferenza, Foggia, Italy

**Keywords:** ALK, RT-qPCR, inverse prediction, FFPE NSCLC, cytology specimens

## Abstract

Accurate detection of altered anaplastic lymphoma kinase (ALK) expression is critical for the selection of lung cancer patients eligible for ALK-targeted therapies. To overcome intrinsic limitations and discrepancies of currently available companion diagnostics for ALK, we developed a simple, affordable and objective PCR-based predictive model for the quantitative measurement of any *ALK* fusion as well as wild-type *ALK* upregulation. This method, optimized for low-quantity/−quality RNA from FFPE samples, combines cDNA pre-amplification with *ad hoc* generated calibration curves. All the models we derived yielded concordant predictions when applied to a cohort of 51 lung tumors, and correctly identified all 17 ALK FISH-positive and 33 of the 34 ALK FISH-negative samples. The one discrepant case was confirmed as positive by IHC, thus raising the accuracy of our test to 100%. Importantly, our method was accurate when using low amounts of input RNA (10 ng), also in FFPE samples with limited tumor cellularity (5–10%) and in FFPE cytology specimens. Thus, our test is an easily implementable diagnostic tool for the rapid, efficacious and cost-effective screening of ALK status in patients with lung cancer.

## INTRODUCTION

The anaplastic lymphoma kinase (ALK) is a transmembrane receptor tyrosine kinase involved in the pathogenesis of different types of human cancers, including anaplastic large-cell lymphoma, neuroblastoma and non-small cell lung cancer (NSCLC) [1–4]. In NSCLC, *ALK* is rearranged in approximately 3–7% of all patients [4–7], and in 20–30% of the subset of lung adenocarcinoma patients who are young and non-/light smokers [6–8].

*ALK* (2p23) rearranges primarily with the echinoderm microtubule-associated protein-like 4 (*EML4*, 2p21) in NSCLC leading to the expression of the *EML4-ALK* fusion oncogene [4, 5]. Several *EML4-ALK* fusion variants have been identified in NSCLC [9], as well as other less-frequent *ALK* translocations involving different fusion partners [5, 10–14]. While *ALK* expression is negligible in the normal lung adult tissue [4], these genetic rearrangements lead to the constitutive expression of chimeric proteins comprised of the kinase domain-containing C-terminus of ALK fused to the N-terminus of the translocation partner, which directs ligand-independent dimerization and activation of ALK [4, 5]. In addition to *ALK* rearrangements, *ALK* gene amplification has also been detected in NSCLC [7, 15, 16], which might represent an additional mechanism of ALK activation, although its clinical significance is yet to be determined [15].

The identification of *ALK* translocations in NSCLC has opened the door to the use of targeted therapies for the treatment of these lung cancers. Crizotinib is a well-tolerated first generation ALK inhibitor [17, 18] that has been shown to be superior to standard chemotherapy both as a first- and second-line treatment [19, 20], while second generation ALK inhibitors, such as alectinib and ceritinib, are effective not only in crizotinib-naïve patients, but also in those patients with acquired resistance to crizotinib [21–24]. The availability of these targeted therapies has prompted the development of diagnostic assays and algorithms that can accurately identify ALK-positive lung cancers patients. Although several methodologies have been developed, they display discrepant results [25–31] and often have limited applicability to formalin-fixed, paraffin-embedded (FFPE) tissue samples, the major source material for diagnostic testing [25]. Thus, a consensus on the optimal technique and testing algorithm has not yet been reached in the clinical setting [25, 32].

Two leading FDA approved ALK diagnostic tests are the break-apart fluorescence *in situ* hybridization (FISH) assay (Vysis ALK Break Apart FISH Probe Kit), and the automated immunohistochemistry (IHC) assay (Ventana ALK (D5F3) CDx Assay). These two tests show a good level of correlation [33] and in a recent international interpretation study they demonstrated an overall sensitivity, specificity and accuracy of 90%, 95% and 93%, respectively [34]. However, the use of alternative approaches, i.e. reverse transcription-polymerase chain reaction (RT-PCR) and/or next-generation sequencing (NGS), has been recommended to resolve discordant or borderline cases [35, 36].

Real-time PCR represents a more quantitative and sensitive technology with reduced inter-observer variability, when compared with FISH and IHC. Yet, some limitations prevent its full implementation in the clinical setting. Firstly, established multiplex RT-PCR assays for the detection of all the different *ALK* rearrangements require continuous optimization, given the increasing numbers of fusion variants and partners identified [37, 38]. Secondly, more recent reverse transcription quantitative real-time PCR (RT-qPCR) assays based on the unbalanced expression of the 5′ and 3′ portions of the *ALK* transcript [39–41], which occurs when *ALK* is rearranged, require significant amounts of RNA (50–100 ng per PCR reaction) from FFPE tissues [40, 41]. Alternative technologies that could be applied to the detection of *ALK* rearrangements, i.e., NanoString (NanoString Technologies, Inc., Seattle, WA) and RNA massive parallel sequencing require, in addition to elevated amounts of total RNA, the availability of proprietary and cutting-edge platforms in pathology laboratories [42, 43]. To circumvent these problems, we describe herein, a simple quantitative PCR-based ALK predictive model fully optimized to work with low-quantity and low-quality RNA from FFPE samples. The test, by targeting both the 5′ and 3′ portions of *ALK* mRNA, detects any *ALK* translocation as well as overexpression of full-length *ALK*.

## RESULTS

### Optimization of the RT-qPCR assay for the detection of *ALK* alterations in FFPE NSCLC samples

**Figure 1:**
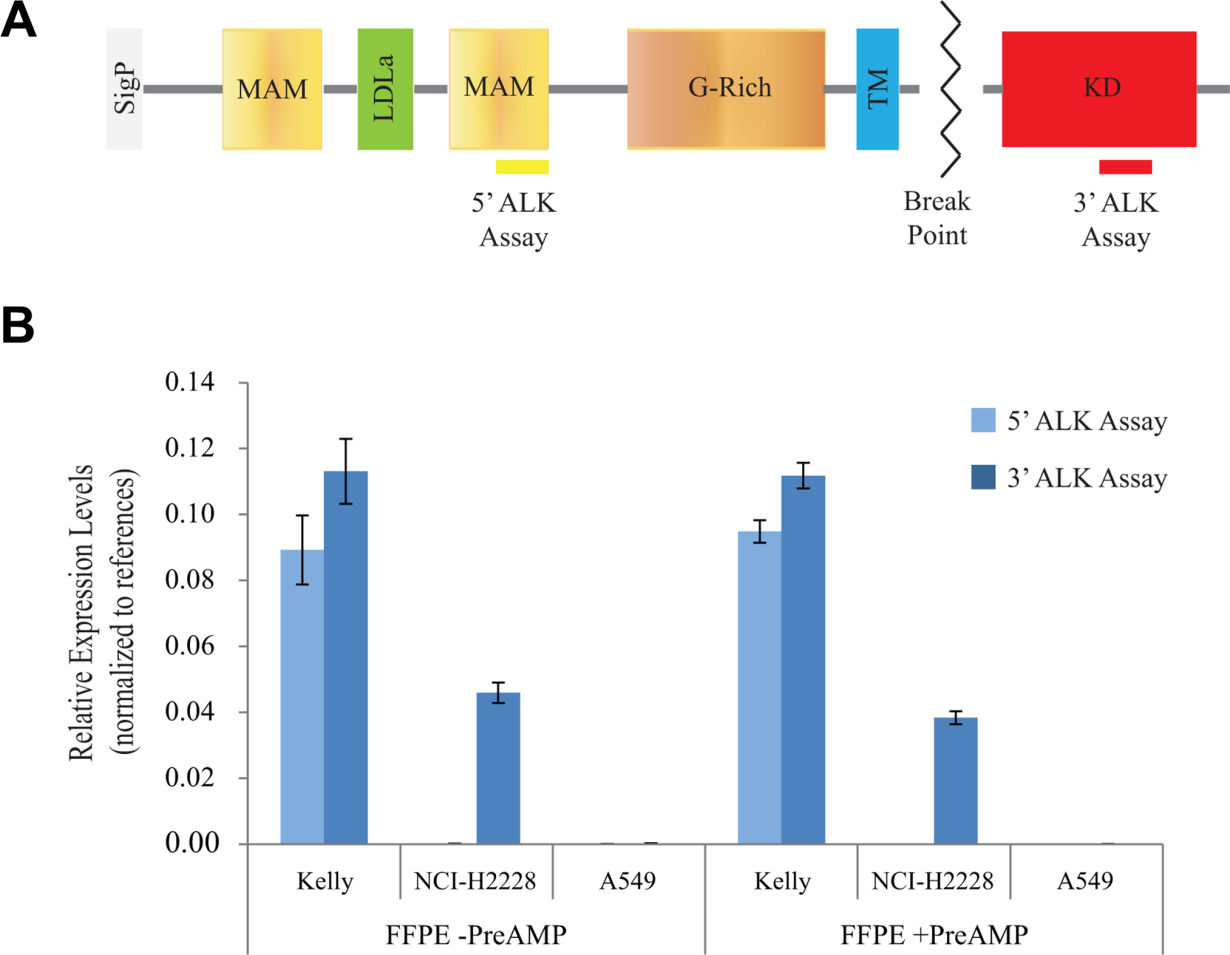
Optimization of the RT-qPCR assays for the detection of the *ALK* transcript (**A**) The position of the two different ALK PCR assays relative to the domain structure of the ALK protein is shown. SigP, signal peptide; MAM (meprin, A-5 protein, and receptor protein-tyrosine phosphatase mu) domains; LDLa (Low-density lipoprotein receptor domain class A) domain; G-rich domain (glycine-rich); TM (Transmembrane) domain; KD (kinase domain). The *ALK* breakpoint that leads to fusion proteins in different cancers is indicated (zigzag line). (**B**) Relative expression levels of the 5′ and 3′ portions of the *ALK* transcript calculated using the formula 2^−ΔC^^q^ (where ΔCq = Cq_ALK_ − average Cq_REF_) in FFPE Kelly, NCI-H2228, and A549 cells with (+ PreAMP) or without (− PreAMP) pre-amplification are reported. Error bars indicate 95% confidence intervals (three independent experiments, *n* = 3).

The breakpoint of *ALK* occurs, by and large, before its intracellular kinase domain (exon 20). When *ALK* is translocated, its C-terminal portion (exons 20–29) is consistently expressed in the chimeric transcript while its N-terminal part (exons 1–19) is lost. Therefore, measuring the unbalanced expression of the C- and N-terminal portions of the *ALK* transcript in a given sample is a reliable method to indirectly identify *ALK* rearrangements, regardless of fusion partner and variant type. Based on this rationale, we selected two different RT-qPCR assays, one targeting exons 27–28 in the 3′ region and the other targeting exons 9–10 in the 5′ region of *ALK* (Figure 1A). In this way, our test is able to detect both known and unknown *ALK* fusions as well as wild-type *ALK* upregulation.

To optimize the RT-qPCR analysis of *ALK* expression from limited amounts of degraded RNA from FFPE tissues, we selected probes targeting short regions (< 90 bp in size) of the transcript to increase the probability of detection. We also implemented a multiplex pre-amplification method designed for the dual purpose of stretching precious sample material into more qPCR reactions and of improving the signal-to-noise ratio for the detection of low/moderate-abundance transcripts.

To test the specificity of the two ALK assays, we employed RNA from FFPE samples of: i) Kelly cells expressing readily detectable levels of full-length *ALK* transcript, containing both the 3′ and 5′ portions of *ALK* mRNA [2]; ii) NCI-H2228 cells expressing an *EML4*-*ALK* translocation, thus positive only for the 3′ portion of the *ALK* mRNA [5]; iii) A549 cells expressing barely detectable levels of the normal *ALK* transcript, used as a negative control [5].

As expected, the 3′ assay detected *ALK* expression levels only in Kelly and NCI-H2228 cells, while the 5′ ALK assay was positive only in Kelly cells (Figure 1B). When the assays were tested on fresh-frozen (FF) samples of the above cell lines and compared with the results from the FFPE samples, similar expression patterns were detected (Supplementary Table S1). Importantly, the relative expression patterns of both the 3′ and 5′ portions of the *ALK* transcript were comparable in the three FFPE cell lines with and without pre-amplification, indicating an equal and efficient pre-amplification of cDNA for all the selected targets (Figure 1B and Supplementary Table S1). Indeed, the mean pre-amplification uniformity values (ΔΔCq) relative to the two ALK assays, measured in Kelly cells were largely within the ± 1.5 value that is generally accepted for uniform pre-amplification reactions (5′ ALK ΔΔCq: − 0.09 ± 0.20; 3′ ALK ΔΔCq: 0.01 ± 0.14; Supplementary Table S2). In addition, pre-amplification resulted in mean Cq improvements of around 8 cycles: e.g., 7.85 ± 0.13 cycles (range: 7.64−7.99) in Kelly cells (see Supplementary Table S3 for a complete analysis of the 3 cell lines).

### Development of an accurate model to predict ALK expression

To predict accurately the expression levels of the 5′ and 3′ portions of *ALK* in NSCLC samples, we used the calibration curves generated with RNA from Kelly and NCI-H2228 cells (see details in Materials and Methods). The curves were used to develop different predictive models, based on an inverse prediction approach [44], for the quantitative assessment of the 3′ and 5′ or only the 3′ portion of the *ALK* transcript.

Initially, we compared the calibration curves prepared with Kelly cells in the presence or absence of pre-amplification. The two linear fits and associated residual sum of squares (RSS) of the data points relative to the 3′ and 5′ *ALK* portions, obtained with these two calibration curves, were comparable (3′ ALK −/+PreAMP, *R*^2^ = 0.99/0.99, RSS = 1.17/1.23; 5′ ALK −/+PreAMP, *R*^2^ = 0.98/0.98, RSS = 1.97/1.46, Supplementary Figure S1A–B). Moreover, Cq improvements of ~7.57 ± 0.40 cycles for the 5′ and ~7.38 ± 0.35 cycles for the 3′ portion of *ALK* across all the data points of the dilution range were retained (Supplementary Table S4). To control for the balanced expression of the 3′ and 5′ portions of *ALK*, we calculated the difference between the ΔCq of the 3′ and of the 5′ portion of *ALK* (ΔΔCq_3′–5′ALK_ = ΔCq_3′ALK_-ΔCq_5′ALK_), relative to the endogenous controls, for each data point of the two calibration curves, in the presence and absence of pre-amplification. These ΔΔCq values are expected to remain stable across the dilution range in the case of balanced 3′/5′ *ALK* expressions. ΔΔCq values were more stable in the pre-amplified (range = − 0.36 − 0.03; median = − 0.10; Q1 = − 0.15; Q3 = − 0.03) *vs.* not pre-amplified (range = − 0.91 − 0.38; median = − 0.30; Q1 = − 0.41; Q3 = − 0.22) calibration curve (Supplementary Figure S1C). Based on these results, pre-amplification was included in the generation of the calibration curve for NCI-H2228 cells, which behaved similarly to Kelly cells (Supplementary Figure S1D).

**Figure 2:**
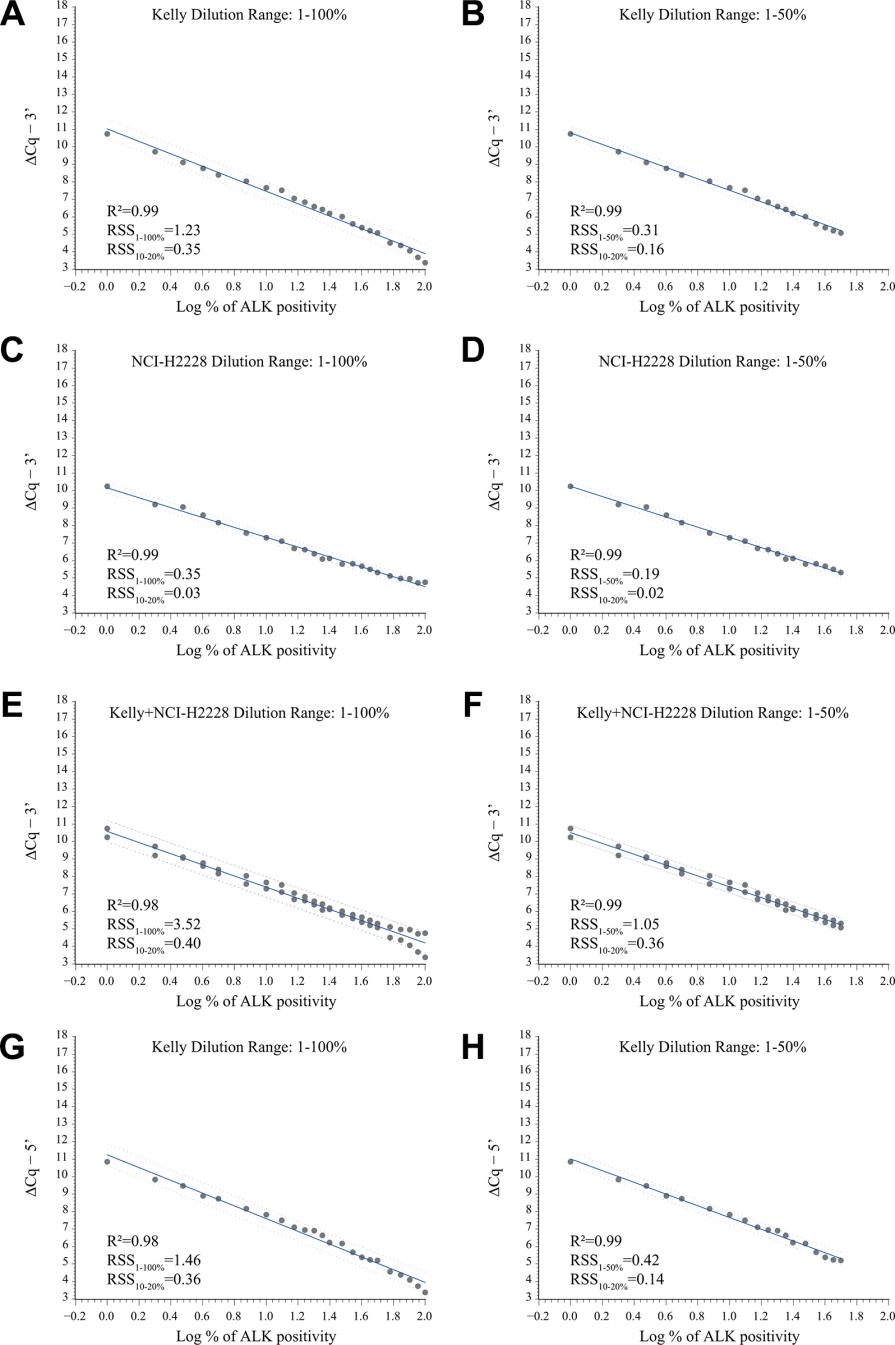
Development of various ALK models using external calibration curves (**A**–**F**) Correlation plots of the expression values (grey dots) of the 3′ portion of *ALK* relative to the 1–100% and the 1–50% dilution range in Kelly (A and B), NCI-H2228 (C and D), and pooled Kelly + NCI-H2228 (E and F) calibration curves. (**G**–**H**) Correlation plots of the expression values (grey dots) of the 5′ portion of *ALK* relative to the 1–100% (G) or the 1–50% (H) dilution range in the Kelly calibration curves. For all curves, linear fit (solid blue line) and 95% prediction limits (dashed blue line) are reported. Relative expression values of the 3′ (ΔCq−3′) or the 5′ (ΔCq−5′) portion of *ALK* normalized to internal controls (ΔCq = Cq_ALK_ − average Cq_REF_) by known percentage (log-transformed, base 10, Log %) of total RNA of *ALK* positive cells in the different calibration curves are indicated. R^2^, coefficient of determination; RSS, residual sum of squares relative to the 1–100% (RSS_1–100__%_), the 1–50% (RSS_1–50__%_) and the 10–20% (RSS_10–20__%_) dilution series, are shown.

Next, we derived different fitting models using the Kelly and NCI-H2228 calibration curves, either by covering the entire 1–100% dilution range or by restricting the analysis to the 1–50% dilution range, where a better sensitivity of the assay is desirable. For the 3′ portion of *ALK*, we used both the Kelly and NCI-H2228 calibration curves, separately and pooled, as they are representative of both *ALK* full-length expression and translocation. For the 5′ portion of *ALK* we used only the curves obtained with Kelly cells. Overall, the different linear fits (6 for the 3′ and 2 for the 5′ *ALK*) were similar (R^2^ range = 0.98–0.99; Figure 2). Parameters relative to the various derived algorithms (Supplementary Table S5) were then used to convert *ALK* transcript expression measured in NSCLC samples by RT-qPCR into percentages of 3′ and/or 5′ *ALK* transcript positivity, as described in the following section.

### Validation of the ALK predictive model in a cohort of NSCLC patients

To define the threshold value for *ALK* positivity (abnormal *ALK* expression) in NSCLC samples, we applied the different predictive models to a set of 20 lung FFPE non-pathological tissue samples. Based on the low basal levels of 3′ *ALK* and barely detectable levels of 5′ *ALK*, measured in non-pathological lung tissues, the threshold value of *ALK* positivity was set at 10% and 3% for the 3′ and 5′ portions, respectively (Supplementary Tables S6A–S6B).

**Figure 3:**
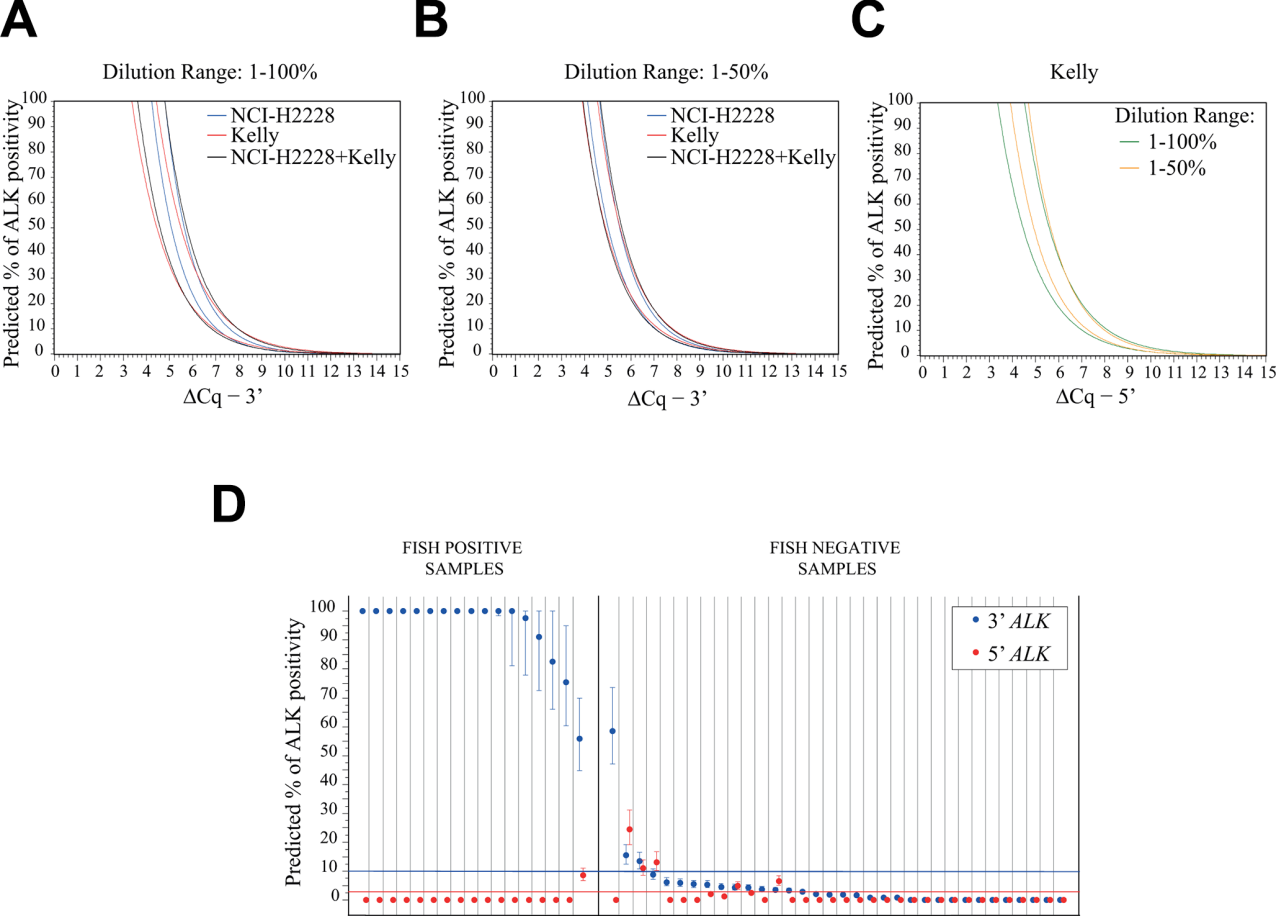
Comparison of the confidence bands in the different models and validation of the Kelly 1–50% ALK predictive model using FFPE NSCLC samples (**A**–**B**) Confidence bands at 95% for predicted percentages of 3′ *ALK* positivity relative to the calibration curves in the 1–100% (A) or the 1–50% (B) range with NCI-H2228 (blue lines), Kelly (red lines) and Kelly + NCI-H2228 (black lines) for the *ALK* 3′ portion. (**C**) Confidence bands at 95% for predicted percentages of 5′ *ALK* positivity specific to the Kelly calibration curves in the 1–100% (green line) or the 1–50% (orange line) range. ΔCq data were simulated ranging from 0 to 15, every 0.01 units. Estimates were obtained through inverse prediction models considering the indicated calibration curves and dilution series. (**D**) Predicted percentage (and 95% confidence bars) of 3′ (blue dots) and 5′ (red dots) *ALK* portions for the 17 FISH-positive and 34 FISH-negative NSCLC samples. Blue and red lines indicate the threshold for the 3′ (10%) and 5′ (3%) *ALK* positivity, respectively.

Next, we applied the derived models to predict *ALK* status in a cohort of 51 NSCLC FFPE samples, previously characterized by FISH for *ALK* translocation, composed of 17 FISH positive and 34 FISH negative samples (Table 1). Of note, we obtained RT-qPCR data of adequate quality for all 51 FFPE NSCLC samples, as determined using the interquartile rule for outliers (see Materials and Methods and Supplementary Table S7). All models gave 100% concordant binary predictions (*ALK* translocated or not translocated) for all the 51 FFPE NSCLC samples, with minor differences in the percentage of 3′ and/or 5′ *ALK* positivity (Supplementary Table S8). Moreover, models generated in the 1–50% dilution range exhibited narrower 95% confidence intervals for all the predicted percentages of 3′ and 5′ *ALK* positivity (including those > 50%), compared with the models derived from the 1–100% dilution range (ΔCq data were simulated ranging from 0 to 15, every 0.01 units, as shown in Figure 3A–3C). No significant differences were observed between the predictions obtained with the Kelly and the NCI-H2228 models in the 1–50% dilution range (Figure 3B). Thus, we used the algorithms generated with the Kelly model, which allows the concomitant detection of both the 3′ and 5′ portions of *ALK*, to predict *ALK* status in the NSCLC cohort (Figure 3D).

**Table 1:** Clinico-pathological characteristics of the NSCLC cohort composed of 51 surgical and 7 cytology specimens analyzed for *ALK* expression

Case ID	Age (yrs)	Sex	Histology	EGFR/KRAS Mutation	Tumor Site (Primary/Metastasis)	Specimen Analyzed	FISH ALK
1	54	F	ADK	NEG/NEG	Metastasis	Pleura	POS
2	65	F	ADK	NEG/NEG	Primary	Lung	POS
3	48	F	ADK	NEG/NEG	Metastasis	Pleura	POS
4	47	M	ADK	ND/ND	Metastasis	Lymph node	POS
5	81	F	ADK	ND/NEG	Primary	Lung	POS
6	66	M	ADK	NEG/NEG	Primary	Lung	POS
7	38	F	ADK	NEG/NEG	Primary	Lung	POS
8	58	M	ADK	NEG/NEG	Metastasis	Lymph node	POS
9	38	F	ADK	NEG/NEG	Metastasis	Lymph node	POS
10	49	F	ADK	NEG/NEG	Metastasis	Lymph node	POS
11	47	F	ADK	NEG/NEG	Metastasis	Pleura	POS
12	52	F	ADK	NEG/NEG	Primary	Lung	POS
13	61	M	ADK	NEG/NEG	Metastasis	Pleura	POS
14	51	F	ADK	NEG/NEG	Metastasis	Lymph node	POS
15	66	M	ADK	NEG/NEG	Metastasis	Pleura	POS
16	36	F	ADK	NEG/NEG	Primary	Lung	POS
17	44	F	ADK	ND/ND	Primary	Lung	POS
18	61	M	ADK	POS/NEG	Metastasis	Pleura	NEG
19	54	F	ADK	NEG/NEG	Primary	Lung	NEG
20	70	M	ADSK + NET	NEG/NEG	Primary	Lung	NEG
21	68	M	ADK	POS/NEG	Primary	Lung	NEG
22	61	M	ADK	NEG/POS	Primary	Lung	NEG
23	63	M	ADK	NEG/NEG	Primary	Lung	NEG
24	56	F	PC	NEG/POS	Primary	Lung	NEG
25	65	F	ADK	NEG/POS	Primary	Lung	NEG
26	58	M	ADK	NEG/NEG	Primary	Lung	NEG
27	63	M	ADK	NEG/POS	Primary	Lung	NEG
28	80	M	ADK	NEG/POS	Primary	Lung	NEG
29	58	F	ADSK	NEG/NEG	Primary	Lung	NEG
30	49	M	ADK	POS/NEG	Metastasis	Pleura	NEG
31	68	M	ADK	NEG/ND	Primary	Lung	NEG
32	71	M	ADK	NEG/ND	Primary	Lung	NEG
33	65	M	ADK	NEG/NEG	Primary	Lung	NEG
34	50	F	ADK	NEG/ND	Primary	Lung	NEG
35	53	M	ADK	NEG/POS	Primary	Lung	NEG
36	53	F	ADK	NEG/ND	Primary	Lung	NEG
37	59	M	ADK	NEG/NEG	Primary	Lung	NEG
38	78	M	ADSK	NEG/ND	Primary	Lung	NEG
39	68	F	ADK	NEG/ND	Primary	Lung	NEG
40	68	M	ADK	NEG/NEG	Primary	Lung	NEG
41	60	M	ADK	NEG/POS	Primary	Lung	NEG
42	59	M	ADK	NEG/ND	Primary	Lung	NEG
43	61	F	ADK	NEG/ND	Primary	Lung	NEG
44	71	M	ADK	NEG/POS	Metastasis	Pleura	NEG
45	62	F	ADK	NEG/POS	Primary	Lung	NEG
46	66	M	ADK	NEG/POS	Metastasis	Pleura	NEG
47	46	F	SCC	POS/NEG	Metastasis	Parietal Pleura	NEG
48	63	M	ADK	POS/NEG	Metastasis	Lymph node	NEG
49	73	F	ADK	NEG/NEG	Metastasis	Lymph node	NEG
50	72	M	ADK	NEG/POS	Metastasis	Pleura	NEG
51	50	M	ADK	NEG/POS	Primary	Lung	NEG
52	55	F	ADK	NEG/NEG	Metastasis	Lymph node/Cytology	NEG
53	82	M	ADK	NEG/NA	Metastasis	Lymph node/Cytology	NEG
54	68	M	ADK	NEG/POS	Metastasis	Lymph node/Cytology	NEG
55	64	M	ADK	NEG/NEG	Metastasis	Lymph node/Cytology	NEG
56	63	M	ADK	NEG/NA	Metastasis	Lymph node/Cytology	NEG
57	75	M	ADK	NEG/POS	Metastasis	Lymph node/Cytology	POS
58	56	F	ADK	NEG/NEG	Metastasis	Lymph node/Cytology	POS

Case IDs #1–51 refer to surgical specimens and Case IDs #52–58 refer to cytology specimens obtained by EBUS. Age at surgery is indicated (yrs); F, female; M, male; ADK, adenocarcinoma; ADSK, adenosquamous cell carcinoma; ADSK + NET, adenosquamous cell carcinoma + neuroendocrine; SCC, squamous; PC, pleomorphic carcinoma of the lung; EGFR, epidermal growth factor receptor; KRAS, Kirsten rat sarcoma viral oncogene homolog; POS, positive; NEG, negative; ND, not determined or missing data; ALK, anaplastic lymphoma kinase; FISH, fluorescent *in situ* hybridization.

Our model identified all the 17 *ALK* FISH-positive samples as translocated. Indeed, all these samples showed an elevated percentage of 3′ positivity compared with the percentage of 5′ positivity, thereby confirming the presence of an unbalanced expression of the two *ALK* regions as a consequence of a translocation event (Table 2). Among the 34 FISH-negative cases, 33 resulted not translocated by our method, while one sample was clearly detected as translocated (Case ID #19, 3′ *ALK* positivity of 58.6%; 95% CI: 47–73.5), despite its scarce tumor cellularity (5%, Table 2). This sample, with a marginal percentage of FISH positive nuclei (10%; Table 2), was ALK-positive by IHC analysis using the automated IHC Ventana assay, in agreement with our PCR assay. Thus, these data indicate that the discrepant FISH result was a false negative and, importantly, that our test reliably identifies *ALK* translocations in specimens with limited tumor cellularity, as low as 5–10% (Case ID #13, #15 and #19, Table 2).

**Table 2:** Accuracy of the PCR-based predictive model in the assessment of ALK status in the 51 NSCLC cohort

Case ID	FISH		RT-qPCR Model - Kelly (1–50%) 100 ng RNA			Tumor Cell Content (%)	Tumor Site (Primary/Metastasis)	Specimen Analyzed
	ALK score	Positive Nuclei (%)	*ALK* Status	3′ *ALK* Positivity (%)	5′ *ALK* Positivity (%)			
1	POS	90	T	82.6 (66.0–100)	0	30	Metastasis	Pleura
2	POS	64	T	100 (100–100)	0	60	Primary	Lung
3	POS	87	T	100 (100–100)	0	60	Metastasis	Pleura
4	POS	61	T	100 (81.1–100)	0	80	Metastasis	Lymph node
5	POS	55	T	100 (100–100)	0	80	Primary	Lung
6	POS	61	T	100 (98.4–100)	0	70	Primary	Lung
7	POS	70	T	100 (100–100)	0	60	Primary	Lung
8	POS	50	T	100 (100–100)	0	60	Metastasis	Lymph node
9	POS	50	T	100 (100–100)	0	60	Metastasis	Lymph node
10	POS	53	T	100 (100–100)	0	70	Metastasis	Lymph node
11	POS	31	T	75.4 (60.3–95.0)	0	20	Metastasis	Pleura
12	POS	35	T	100 (100–100)	0	30	Primary	Lung
13	POS	23	T	97.7 (77.8–100)	0	10	Metastasis	Pleura
14	POS	24	T	91.1 (72.6–100)	0	40	Metastasis	Lymph node
15	POS	32	T	55.8 (44.8–69.9)	8.7 (6.8–11.0)	5	Metastasis	Pleura
16	POS	24	T	100 (100–100)	0	50	Primary	Lung
17	POS	25	T	100 (100–100)	0	60	Primary	Lung
18	NEG	8	NT	0	0	70	Metastasis	Pleura
19	NEG	10	T	58.6 (47.0–73.5)	0	5	Primary	Lung
20	NEG	3	NT	13.5 (10.9–16.6)	11.0 (8.6–14.0)	80	Primary	Lung
21	NEG	0	NT	4.6 (3.7–5.7)	1.2 (0.9–1.5)	70	Primary	Lung
22	NEG	8	NT	6.2 (5.0–7.7)	0	60	Primary	Lung
23	NEG	2	NT	3.8 (3.1–4.8)	0	80	Primary	Lung
24	NEG	5	NT	0.8 (0.6–1.0)	0	60	Primary	Lung
25	NEG	11	NT	15.5 (12.5–19.2)	24.4 (19.2–31.3)	50	Primary	Lung
26	NEG	0	NT	5.5 (4.4–6.8)	0	30	Primary	Lung
27	NEG	5	NT	4.4 (3.6–5.5)	4.9 (3.8–6.3)	30	Primary	Lung
28	NEG	0	NT	3.3 (2.7–4.1)	0	60	Primary	Lung
29	NEG	1	NT	1.8 (1.4–2.2)	0	80	Primary	Lung
30	NEG	4	NT	0	0	70	Metastasis	Pleura
31	NEG	3	NT	2.0 (1.6–2.5)	0	80	Primary	Lung
32	NEG	3	NT	5.4 (4.4–6.7)	2.0 (1.5–2.5)	80	Primary	Lung
33	NEG	0	NT	0	0	80	Primary	Lung
34	NEG	4	NT	8.8 (7.1–10.9)	13.1 (10.3–16.7)	90	Primary	Lung
35	NEG	0	NT	0	0	60	Primary	Lung
36	NEG	0	NT	0	0	60	Primary	Lung
37	NEG	5	NT	0	0	80	Primary	Lung
38	NEG	5	NT	0	0	70	Primary	Lung
39	NEG	2	NT	4.3 (3.4–5.3)	2.4 (1.9–3.1)	70	Primary	Lung
40	NEG	2	NT	2.9 (2.3–3.6)	0	60	Primary	Lung
41	NEG	4	NT	1.7 (1.4–2.1)	0	60	Primary	Lung
42	NEG	13	NT	0	0	40	Primary	Lung
43	NEG	6	NT	0	0	80	Primary	Lung
44	NEG	13	NT	0.9 (0.7–1.1)	0	80	Metastasis	Pleura
45	NEG	1	NT	0	0	40	Primary	Lung
46	NEG	3	NT	3.6 (2.9–4.4)	6.5 (5.1–8.3)	5	Metastasis	Pleura
47	NEG	4	NT	0.9 (0.7–1.1)	0	70	Metastasis	Parietal Pleura
48	NEG	2	NT	6.0 (4.8–7.4)	0	40	Metastasis	Lymph node
49	NEG	1	NT	1.8 (1.4–2.2)	0	40	Metastasis	Lymph node
50	NEG	4	NT	0	0	20	Metastasis	Pleura
51	NEG	1	NT	0	0	60	Primary	Lung
Kelly	–	–	NT	100 (100–100)	100 (100–100)	–	–	–
NCI-H2228	–	–	T	71.8 (57.4–90.3)	0	–	–	–
A549	–	–	NT	0	0	–	–	–

Binary *ALK* status by FISH analysis is indicated as positive (POS, ≥ 15% of positive nuclei) or negative (NEG, < 15% of positive nuclei). Percentage (%) of FISH positive nuclei with separated green and red signals or single red signals (translocation and deletion of the *ALK* 5′ portion, respectively) are reported when available. Binary *ALK* status determined by the PCR-based model analysis is indicated as Translocated (T) or Not Translocated (NT) based on the predicted percentage of 3′ and 5′ *ALK* positivity measured in each FFPE NSCLC sample (positivity cut-off value for the 3′ and 5′ *ALK* portions were 10% and 3%, respectively). Predicted percentages of 3′ and 5′ *ALK* positive RNA present in FFPE NSCLC samples are reported along with the 95% confidence intervals in brackets. These values were calculated from the data obtained in the RT-qPCR analysis by the ALK inverse prediction models obtained from the Kelly calibration curves for the 3′ and 5′ portions of *ALK*. The model was derived considering the 1–50% dilution series of the external calibration curves. *ALK* transcript expression was measured using the PCR protocol optimized for FFPE samples starting from 100 ng of input RNA. The percentage (%) of tumor cell content is also indicated for each sample. ALK, anaplastic lymphoma kinase; FISH, fluorescence *in-situ* hybridization; RT-qPCR, quantitative reverse transcription-polymerase chain reaction.

Among the 33 tumors identified as not translocated by our PCR assay, two exhibited a level of 3′ positivity significantly above the 10% cut-off (Case ID #20, 13.5%; Case ID #25, 15.5%, Table 2). Both these samples, however, showed percentages of 5′ positivity comparable to the 3′ portion (Table 2), suggesting that they did not harbor an *ALK* translocation, but rather expressed low/moderate levels of normal *ALK* transcripts. Together, these results showed that our diagnostic model reached a sensitivity, specificity and accuracy of 100%, 97% and 98%, respectively, compared with FISH.

To evaluate whether our predictive model was accurate when using low quantities of input RNA, representative FFPE NSCLC samples (21 in total), covering a wide range of *ALK* positivity, were processed using 25 and 10 ng of total RNA instead of 100 ng. The predictions relative to the three different RNA quantities (100, 25 and 10 ng) were 100% concordant, indicating that our model reliably detects *ALK* status in FFPE NSCLC samples also when the input RNA is scarce (Table 3).

**Table 3:** Accuracy of *ALK* status prediction in a selected subset of 21 NSCLC samples using 100, 25 and 10 ng of input RNA

Case ID	FISH		RT–qPCR Model - Kelly (1–50%)									Tumor Cell Content (%)
			100 ng RNA			25 ng RNA			10 ng RNA			
	ALK Score	Positive Nuclei (%)	*ALK* Status	3′ *ALK* Positivity (%)	5′ *ALK* Positivity (%)	*ALK* Status	3′ *ALK* Positivity (%)	5′ *ALK* Positivity (%)	*ALK* Status	3′ *ALK* Positivity (%)	5′ *ALK* Positivity (%)	
5	POS	55	T	100 (100–100)	0	T	100 (100–100)	0	T	100 (100–100)	0	80
6	POS	61	T	100 (98.4–100)	0	T	100 (82.8–100)	0	T	96.4 (76.8–100)	0	70
7	POS	70	T	100 (100–100)	0	T	100 (100–100)	0	T	100 (100–100)	0	60
8	POS	50	T	100 (100–100)	0	T	100 (100–100)	0	T	100 (100–100)	0	60
9	POS	50	T	100 (100–100)	0	T	100 (100–100)	0	T	100 (100–100)	0	60
11	POS	31	T	75.4 (60.3–95.0)	0	T	83.8 (66.9–100)	0	T	100 (100–100)	0	20
13	POS	23	T	97.7 (77.8–100)	0	T	79.7 (63.7–100)	0	T	85.5 (68.3–100)	0	10
16	POS	24	T	100 (100–100)	0	T	100 (100–100)	0	T	100 (100–100)	0	50
19	NEG	10	T	58.6 (47.0–73.5)	0	T	48.5 (39.0.–60.7)	0	T	63.7 (51.1–80.0)	0	5
20	NEG	3	NT	13.5 (10.9–16.6)	11.0 (8.6–14.0)	NT	10.3 (8.3–12.7)	11.6 (9.1–14.8)	NT	8.9 (7.2–11.0)	9.0 (7.0–11.4)	80
21	NEG	0	NT	4.6 (3.7–5.7)	1.2 (0.9–1.5)	NT	5.1 (4.1–6.3)	1.3 (1.0–1.6)	NT	5.4 (4.4–6.7)	3.0 (2.3–3.9)	70
22	NEG	8	NT	6.2 (5.0–7.7)	0	NT	5.2 (4.2–6.5)	0	NT	4.3 (3.5–5.4)	0	60
23	NEG	2	NT	3.8 (3.1–4.8)	0	NT	3.5 (2.8–4.3)	0	NT	4.7 (3.8–5.9)	0	80
26	NEG	0	NT	5.5 (4.4–6.8)	0	NT	4.4 (3.6–5.5)	0	NT	7.6 (6.2–9.4)	0	30
38	NEG	5	NT	0	0	NT	0	0	NT	0	0	70
39	NEG	2	NT	4.3 (3.4–5.3)	2.4 (1.9–3.1)	NT	6.4 (5.1–7.9)	4.4 (3.4–5.6)	NT	8.1 (6.5–10.0)	0	70
43	NEG	6	NT	0	0	NT	0	0	NT	0	0	80
44	NEG	13	NT	0.9 (0.7–1.1)	0	NT	0	0	NT	0	0	80
48	NEG	2	NT	6.0 (4.8–7.4)	0	NT	5.1 (4.1–6.3)	0	NT	0	0	40
49	NEG	1	NT	1.8 (1.4–2.2)	0	NT	2.6 (2.1–3.2)	0	NT	0	0	40
51	NEG	1	NT	0	0	NT	0	0	NT	0	0	60

Binary *ALK* status by FISH analysis is indicated as positive (POS, ≥ 15% of positive nuclei) or negative (NEG, < 15% of positive nuclei). Percentage (%) of FISH positive nuclei with separated green and red signals or single red signals (translocation and deletion of the *ALK* 5′ portion, respectively) are reported when available. Binary *ALK* status determined by the PCR-based model analysis is indicated as Translocated (T) or Not Translocated (NT) based on the predicted percentage of 3′ and 5′ *ALK* positivity measured in each FFPE NSCLC sample using 100, 25 or 10 ng of RNA input (positivity cut-off values for the 3′ and 5′ *ALK* portions were 10% and 3%, respectively). Predicted percentages of 3′ and 5′ *ALK* positive RNA present in FFPE NSCLC samples are reported along with 95% confidence intervals in brackets. These values were calculated from the data obtained in the RT-qPCR analysis by the ALK inverse prediction models obtained from the Kelly calibration curves for the 3′ and 5′ portions of *ALK*. The model was derived considering the 1–50% dilution series of the external calibration curves. The percentage (%) of tumor cell content is also indicated for each sample. ALK, anaplastic lymphoma kinase; FISH, fluorescence *in situ* hybridization; RT-qPCR, quantitative reverse transcription-polymerase chain reaction.

Finally, we analyzed 7 cytological FFPE samples (5 *ALK* FISH-negative and 2 *ALK* FISH-positive), obtained with the minimally invasive endobronchial ultrasound-guided transbronchial needle aspiration (EBUS-TBNA) procedure, using 10 ng input RNA (Table 1). Our assay correctly identified all the 5 FISH-negative cases and one of the two FISH-positive cases (Case ID #58, with 38% of FISH positive nuclei; Table 4). Notably, the one discordant case (Case ID #57, with a borderline 20% of FISH-positive nuclei) was confirmed to be negative by subsequent IHC analysis.

**Table 4:** Accuracy of *ALK* status prediction in the 7 cytology specimens using 10 ng of input RNA

Case ID	FISH		RT-qPCR Model - Kelly (1–50%) - 10 ng RNA			Tumor Cell Content (%)
	ALK Score	Positive Nuclei (%)	*ALK* Status	3′ *ALK* Positivity (%)	5′ *ALK* Positivity (%)	
52	NEG	11	NT	0	0	60
53	NEG	9	NT	0	0	80
54	NEG	9	NT	0	0	30
55	NEG	12	NT	0	0	50
56	NEG	9	NT	0	14.6 (11.5–18.6)	40
57	POS	20	NT	0	0	70
58	POS	38	T	40.4 (32.5–50.4)	0	70

Binary *ALK* status by FISH analysis is indicated as positive (POS, ≥ 15% of positive nuclei) or negative (NEG, < 15% of positive nuclei). Percentage (%) of FISH positive nuclei with separated green and red signals or single red signals (translocation and deletion of the *ALK* 5′ portion, respectively) are reported. Binary *ALK* status determined by the PCR-based model analysis is indicated as Translocated (T) or Not Translocated (NT) based on the predicted percentage of 3′ and 5′ *ALK* positivity measured in each cytology sample using 10 ng of RNA input (positivity cut-off values for the 3′ and 5′ *ALK* portions were 10% and 3%, respectively). Predicted percentages of 3′ and 5′ *ALK* positive RNA present in FFPE NSCLC samples are reported along with 95% confidence intervals in brackets. These values were calculated from the data obtained in the RT-qPCR analysis by the *ALK* inverse prediction models obtained from the Kelly calibration curves for the 3′ and 5′ portions of *ALK*. The model was derived using the 1–50% dilution series of the external calibration curves. The percentage (%) of tumor cell content is also indicated for each sample. ALK, anaplastic lymphoma kinase; FISH, fluorescence *in situ* hybridization; RT-qPCR, quantitative reverse transcription-polymerase chain reaction.

**Figure 4:**
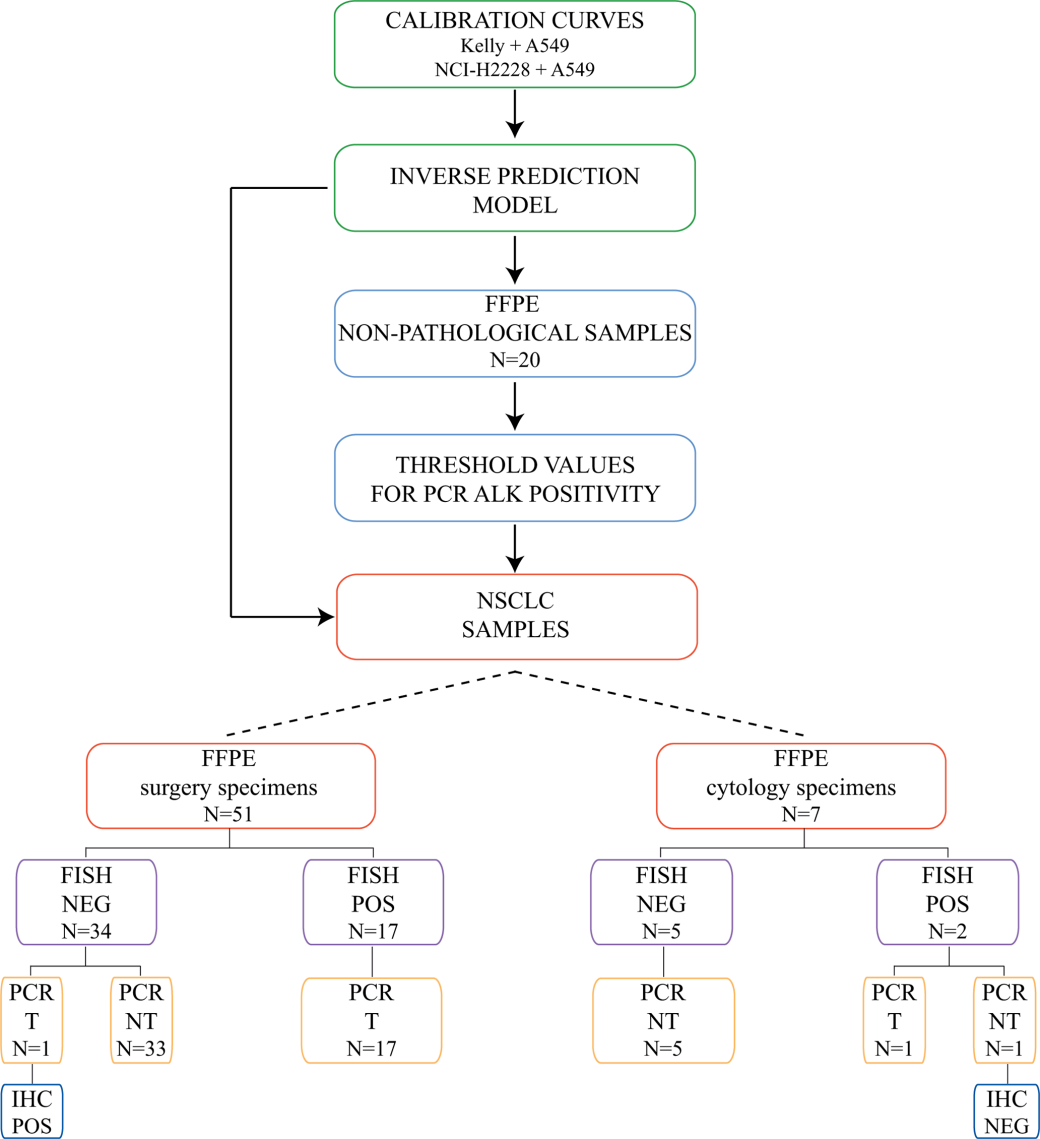
Study design for the development of the RT-qPCR-based prediction model and the validation on 58 FFPE NSCLC tissue specimens Schematic representation of the analysis of *ALK* status in the NSCLC cohort. The cohort was composed of 51 surgical and 7 cytology FFPE NSCLC specimens (see Table 1). Binary *ALK* status by FISH analysis is indicated as positive (POS, ≥ 15% of positive nuclei) or negative (NEG, < 15% of positive nuclei). Binary *ALK* status determined by the PCR-based model analysis is indicated as Translocated (T) or Not Translocated (NT) based on the predicted percentage of 3′ and 5′ *ALK* positivity (cut-off values for 3′ and 5′ *ALK* portions were 10% and 3%, respectively). Binary *ALK* status by IHC is indicated as positive (POS) or negative (NEG) based on the presence or lack of a strong granular cytoplasmic staining in tumor cells, respectively.

In summary, our diagnostic model reached a sensitivity, specificity and accuracy of 95%, 97% and 97%, respectively, compared with FISH on a total of 58 FFPE NSCLC samples (51 surgical and 7 cytology specimens) (Figure 4). Notably, the two discrepant cases (one surgical and one cytological) were confirmed as positive by IHC, thus raising the accuracy of our test to 100%.

## DISCUSSION

The discovery of effective ALK-targeted therapies for the treatment of NSCLC demands that we improve our ability to identify patients eligible for treatment. The FDA-approved break-apart FISH assay represented the gold standard for the detection of *ALK* rearrangements during the crizotinib trials. However, given the difficulty in signal interpretation [45], the FISH assay yields erroneous results [18, 21, 23, 46, 47]. The recently approved Ventana ALK IHC (D5F3) CDx assay is a viable alternative to FISH. Although this IHC assay provides a simple digital reading of ALK status to limit inter-observer variability, a qualified pathologist is needed to grade the result and proprietary stainer and kits are required. Therefore, more quantitative, objective, and open source methodologies could find widespread application in clinical laboratories and help to resolve discrepant cases and/or equivocal results.

Quantitative PCR is a reliable and extremely sensitive technique for the measurement of clinical biomarkers. Optimal PCR-based ALK assays in clinical practice should be i) versatile (able to detect any ALK alteration), ii) accurate (especially when using low quantity, poor quality and low tumor cellularity FFPE biological samples), iii) user-friendly (implementable in routine clinical labs), and iv) cost-effective (particularly relevant for the screening of large, low-incidence populations). Our simple RT-qPCR-based predictive model fulfills these requirements. The combination of a simple inverse prediction model with an optimized RT-qPCR protocol showed that our ALK test was superior to the break-apart FISH assay in a cohort of 58 FFPE NSCLC cases, composed of 51 surgical and 7 cytology specimens. Overall, our diagnostic model reached a sensitivity, specificity and accuracy of 95%, 97% and 97%, respectively, compared with FISH (Tables 2 and 4, Figure 4), and further validation on a larger cohort is now merited. Notably, the accuracy of our test rose to 100% when the FISH false-negative surgical sample and the FISH false-positive cytology sample were reclassified as ALK-positive and ALK-negative, respectively, according to IHC (Figure 4). Our predictive test also provided high percentages of 3′ *ALK* positivity in samples with borderline percentages of FISH-positive nuclei (range = 10–35%), demonstrating superior sensitivity (Table 2 and Table 3). Additionally, our assay was accurate when using as little as 10 ng of input RNA, also in samples with low tumor cellularity (5–10%, Tables 2 and 3) and in cytological specimens (Table 4), which is frequently the only clinical material available in patients with advanced lung cancer. Notably, in a couple of cases (Case ID #20 and #25, Table 2), our test detected overexpression of full-length *ALK*, in the absence of *ALK* translocation (Table 2). Whether this higher expression identifies a small fraction of lung cancers sensitive to ALK inhibitors remains to be established. In addition, by targeting exons 27–28 in the 3′ portion of *ALK*, our assay is able to detect also the recently identified *ALK* isoforms (though rare in lung cancer), encompassing exons 20–29 [48].

Our test offers several advantages over published [39–43] assays assessing the unbalanced expression of the 3′ and 5′ portion of *ALK* transcript: i) it requires at least 5–10 times less input RNA; ii) it does not require expensive and proprietary technologies or specialist expertise (unlike NanoString and/or NGS platforms), and iii) it provides a quantitative assessment of 3′ and 5′ *ALK* mRNA positivity. Unlike traditional comparative (ΔΔCq) and/or 3′/5′ ratio methods derived thereof, our model provides confidence intervals for each point prediction. These interval estimates, by identifying the range of possible true values of the point prediction, will increase clinicians’ confidence about the prediction score and help guide them in therapy decision-making.

Novel multiplex NGS assays represent attractive diagnostic tools for the detection of clinically relevant genomic alterations associated with solid tumors including *ALK* translocations [36, 49]. An extended comparative analysis of our predictive model *vs.* emerging NGS assays, employing large NSCLC cohorts, and technically challenging FFPE specimens is warranted. However, our results clearly show that our PCR-based ALK test is highly accurate in surgical as well as cytological specimens. Therefore, our diagnostic assay not only can be employed as a confirmatory test of FISH and/or IHC results and to resolve equivocal FISH and/or IHC results, but can also be used as a first-line diagnostic tool for the rapid, efficacious and convenient screening of large patient populations.

## MATERIALS AND METHODS

### Human cell lines and samples

The human neuroblastoma cell line Kelly (DSMZ^®^ ACC 355™) and the lung cancer cell line NCI-H2228 (ATCC^®^ CRL-5935^™^) were used as positive controls for the expression of full-length *ALK* transcript (ALKF1174L mutant) [2] and of translocated *ALK* (*EML4-ALK*) [5], respectively. The lung cancer cell line A549 (ATCC^®^ CCL-185™) was used as a negative control, since they express barely detectable levels of the normal *ALK* transcript [5]. Cells were cultured and used fresh or pelleted and processed into FFPE cell blocks for subsequent analyses. Cells were routinely tested for mycoplasma contamination [50] and multiplex short tandem repeat profiling test for authentication using the GenePrint^®^ 10 System (Promega Corporation, Madison, WI, USA).

All samples were derived from patients operated at the European Institute of Oncology (IEO), Milan, Italy. FFPE tissue blocks of ALK FISH-positive samples with sufficient biological material were available for 17 patients. Based on the availability of these 17 FISH-positive samples, we randomly selected 34 FISH-negative controls, which together constituted a cohort of 51 NSCLC patients (with a 1:2 ratio of ALK positive *vs.* control samples). We also analyzed 7 cytological samples (cytoblocks), 5 FISH-negative and 2 FISH-positive, with sufficient biological material obtained with the EBUS-TBNA procedure from patients with clinically diagnosed primary lung cancer. The clinico-pathological characteristics of the patients are described in Table 1. RNA was also extracted from FFPE non-pathological lung tissue samples, adjacent to diseased area, prepared from an additional 20 NSCLC patients. Each case was centrally reviewed to confirm the histopathological assessment and to verify the content of tumor or normal parenchyma. Investigations were conducted in accordance with the ethical standards as outlined in the Declaration of Helsinki and in national and international guidelines, and were approved by the IEO institutional review board.

### Fluorescence *in situ* hybridization

*ALK* rearrangements were analyzed on 4 μm thick FFPE tissue sections, using the break-apart probe FISH Probe Kit (Vysis LSI ALK Dual Color, Abbott Molecular Inc.) according to manufacturer's instructions. Samples were analyzed using an epifluorescence microscope (Leica, Wetzlar, Germany). Signal evaluation was performed in at least 60 nuclei as follows: i) separated green and red signals or single red signals (translocation and deletion of the *ALK* 5′ portion) in at least 15% of tumor cells analyzed; ii) overlapping of green and red signals (yellowish) indicated cells in which *ALK* was not rearranged.

### Immunohistochemistry

ALK IHC was performed on 4 μm thick FFPE tissue sections using the fully automated Ventana IHC ALK (D5F3) CDx Assay (Ventana Medical Systems, Inc., Tucson, AZ, USA), with the pre-diluted Ventana anti-ALK (D5F3) rabbit monoclonal primary antibody, the Optiview DAB IHC detection kit and Optiview Amplification kit on the Benchmark XT stainer. We adopted the binary scoring system (positive or negative for ALK status) to evaluate the staining results according to manufacturer's recommendations. ALK positivity was assigned exclusively in the presence of a strong granular cytoplasmic staining in tumor cells (any percentage of positive tumor cells).

### RNA extraction and quantitative real-time PCR

Genetic material was isolated from fresh cell lines using the AllPrep DNA/RNA/miRNA Universal Kit (Qiagen, Hilden, Germany) and from FFPE cell blocks or tissue blocks using the AllPrep DNA/RNA FFPE Kit (Qiagen, Hilden, Germany). RNA was extracted from manually microdissected areas of 2 tissue sections (10 μm thick) on glass slides selected by a pathologist for each relevant FFPE tissue block. For standard mRNA analysis, 500 ng of total RNA (RNA concentration measured using the NanoDrop^®^ ND-1000 Spectrophotometer) were reverse transcribed with random primers using the SuperScript^®^ VILO™ cDNA Synthesis Kit (Thermo Fisher Scientific) and 5 ng or 20 ng of cDNA from fresh or FFPE cells, respectively, were then analyzed per reaction by PCR. In case of pre-amplification, 100, 25, and 10 ng (as indicated) of total RNA from FFPE cell blocks and/or FFPE tissue blocks were reverse transcribed, pre-amplified for 10 cycles using the PreAMP Master Mix Kit (Thermo Fisher Scientific) according to manufacturer's instructions, and diluted 1:5 prior to PCR analysis (5 μl were then used per PCR reaction, corresponding to 2, 0.5 or 0.2 ng of cDNA).

Quantitative PCR was performed with hydrolysis probes (Thermo Fisher Scientific) using the SsoAdvanced Universal Probes Supermix (Bio-Rad Laboratories) in 10 μl of final volume in 384-well plates. PCR reaction was run in LightCycler (LC) 480 real-time PCR instruments (Roche) using the following thermal cycling conditions: 1 cycle at 95°C for 30 sec, 45 cycles at 95°C for 5 sec, and 60°C for 30 sec.

TaqMan gene expression assays, with short amplicon sizes, were as follows: Hs01058323_m1 (human ALK, RefSeq NM_004304, exon boundary 9–10, assay location 2775, amplicon length 66 bp), Hs00608292_m1 (human ALK, RefSeq NM_004304, exon boundary 27–28, assay location 5022, amplicon length 59 bp), Hs03929097_g1 (human GAPDH, RefSeq NM_001256799, exon boundary 8–8, assay location 1250, amplicon length 58 bp), Hs99999908_m1 (human GUSB, RefSeq NM_000181, exon boundary 11–12, assay location 1925, amplicon length 81 bp) and Hs00427621_m1 (human TBP, RefSeq NM_001172085, exon boundary 3–4, assay location 666, amplicon length 65 bp) (sequence details in Supplementary Table S9).

We defined Cq = 40 as our limit of detection in the absence of pre-amplification and Cq = 30 in the presence of the pre-amplification, based on the lowest Cq value measured for the two ALK assays in the negative control cell line A549 (Supplementary Table S1). Cq values beyond these limits were set to 40 or 30, accordingly, and normalization was omitted. Each target was assayed in triplicate and average Cq values were calculated either from triplicate values when the standard deviation was < 0.4, or from the best duplicate values when the standard deviation was ≥ 0.4. In each sample, the average Cq value of the 3′ or 5′ portion of *ALK* (Cq_ALK_) was normalized on the average Cq (Cq_REF_) value of three human reference genes (*GAPDH, GUSB* and *TBP*), to account for variation in the expression of single reference genes and in RNA integrity due to tissue fixation, using the following formula:

$$\Delta \text{Cq}={\text{Cq}}_{\text{ALK}}-\text{mean }{\text{Cq}}_{\text{REF}}$$

Pre-amplification uniformity for each *ALK* gene expression assay was measured in Kelly cells by calculating the ΔCq for each of the two ALK assays (ΔCq = Cq_3′/5′ALK_ − mean Cq_REF_) and by determining the ΔΔCq between pre-amplified (PreAmp) and not-pre-amplified cDNA templates (ΔΔCq = ΔCq_PreAmp_ − ΔCq_cDNA_). Minus-reverse transcriptase (“−RT”) controls were also performed for pre-amplified cDNA templates to confirm the specificity of the ALK assays. “−RT” controls were negative both for the 3′ and 5′ ALK assays (all samples were flagged as “undetectable”). Based on the distribution of the reference genes, we applied the Tukey's interquartile rule for outliers [51] to identify poor quality RT-qPCR data in the cohort of 51 FFPE NSCLC samples.

### Development of the predictive model for the assessment of *ALK* status in tumor specimens

We prepared two different calibration curves using an artificial dilution series of total RNA derived from FFPE blocks of either Kelly cells expressing full-length *ALK* or NCI-H2228 cells expressing *EML4-ALK*, mixed with total RNA from *ALK* negative A549 cells. Each curve was composed of 23 data points, obtained by diluting RNA from positive cells with increasing amounts of RNA from negative cells until a mixture composed of 1% NCI-H2228/Kelly RNA and 99% A549 RNA was reached. The upper half of the curve (50–100% of RNA from positive cells) was obtained using discrete increments of 10% of RNA from negative cells. To improve the resolution of the ALK assay in the lower half of the calibration curve (0–50%), RNA from positive cells was diluted using progressively smaller increments of RNA from negative cells as follows: 5% increments in the 25–50% range, 2.5% increments in the 5–25% range, and 1% increments in the 1–5% range. We reverse transcribed 500 ng or 100 ng of total RNA for each data point of the calibration curve in the absence or in the presence of pre-amplification, respectively, prior to PCR analysis.

The level of expression of the 3′ and/or the 5′ portion of the *ALK* transcript measured by PCR was regressed on the known percentage (log-transformed, base 10) of total *ALK*-positive RNA present in each data point of the calibration curve analyzed. The inverse prediction approach was applied to predict the actual percentage of 3′ and/or 5′ *ALK*-positive transcript, as well as 95% confidence intervals from the values obtained in the RT-qPCR analysis [44].

We employed both Kelly and NCI-H2228 calibration curves, separately or pooled, to build the predictive models for the 3′ portion; the Kelly calibration curve alone was used for the 5′ portion of *ALK*. To improve the performance of the assay when ALK is expressed at low/moderate levels in unknown NSCLC samples, we also generated predictive models by including only the data points in the 1–50% dilution range. Coefficient of determination (*R*^2^) and residual sum of squares (RSS) values were calculated to assess the goodness of the various fits. To evaluate the basal level of expression of both the 5′ and 3′ portions of *ALK*, we applied the different predictive models to the set of 20 lung FFPE non-pathological tissue samples. The threshold value of *ALK* positivity (abnormal *ALK* expression) was established based on the median of the 95th percentiles of the predictions obtained from each of the different models. Finally, these inverse predictive models were validated in the cohort of 51 FFPE human samples. Translocation was assigned based on unbalanced interval estimates of 3′ and 5′ ALK predictions. Statistical analyses were performed using SAS (SAS 9.3, SAS Institute, Cary, NC, USA).

## SUPPLEMENTARY MATERIALS FIGURE AND TABLES


