# Supplementary material for: Sensitive and affordable diagnostic assay for the quantitative detection of anaplastic lymphoma kinase (ALK) alterations in patients with non-small cell lung cancer
Source: Oncotarget. 2016 May 19;7(24):37160–76. doi: 10.18632/oncotarget.9471 (PMC5095066; doi:10.18632/oncotarget.9471)
Supplement: Supplementary file 1 [file oncotarget-07-37160-s001.pdf]

Sensitive and affordable diagnostic assay for the quantitative detection of anaplastic lymphoma kinase (*ALK*) alterations in patients with non-small cell lung cancer

Supplementary Materials

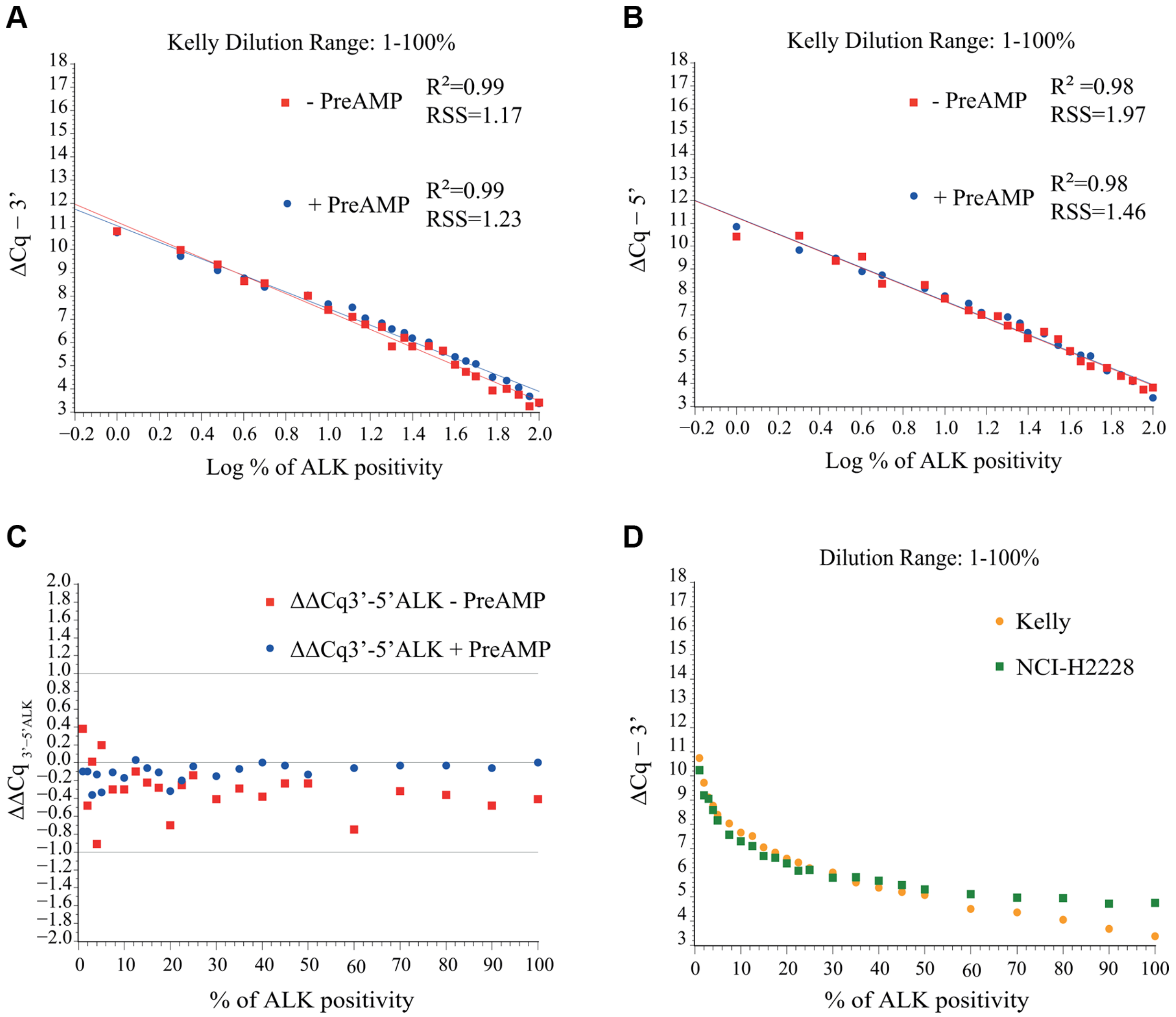

Supplementary Figure S1: Features of the different calibration curves, with and without pre-amplification, in Kelly and NCI-H2228 cells. (**A**, **B**). Correlation plots of the expression values relative to the 3′ (A) and the 5′ (B) portion of *ALK* obtained in Kelly cells with (blue dots) and without (red squares) pre-amplification (1–100% dilution range). Relative expression values of the 3′ (ΔCq−3′) or the 5′ (ΔCq−5′) portion of *ALK* normalized to internal controls ($\Delta Cq = Cq_{ALK} - \text{average } Cq_{REF}$) by known percentage (log-transformed, base 10, Log %) of total RNA of *ALK* positive cells in the different calibration curves are indicated (log % of *ALK* positivity). R^2, coefficient of determination; RSS, residual sum of squares. (**C**). Comparison of the distribution of the ratio 3′–5′ *ALK* ($\Delta\Delta Cq_{3'-5'ALK} = \Delta Cq_{3'ALK} - \Delta Cq_{5'ALK}$) across all the data points of the Kelly cell calibration curves obtained without (red squares) and with (blue dots) pre-amplification. (**D**). Relative expression values of the 3′ (ΔCq−3′) portion of *ALK* in Kelly (orange dots) and NCI-H2228 (green squares) calibration curves (1–100% dilution range).

Supplementary Table S1: Raw Cq values in fresh frozen (FF) and formalin-fixed, paraffin-embedded (FFPE) Kelly, NCI-H2228 and A549 cells

				5′ *ALK* Cq	3′ *ALK* Cq	*GUSB* Cq	*GAPDH* Cq	*TBP* Cq	Mean REFs (GUSB/ GAPDH/ TBP) Cq	$\Delta Cq_{5'ALK}$ ($Cq_{5'ALK}$ - $Cq_{MeanREFs}$)	$\Delta Cq_{3'ALK}$ ($Cq_{3'ALK}$ - $Cq_{MeanREFs}$)	2^- ΔCq5′ ALK	2^- ΔCq3′ ALK
**Replica A**	**Kelly**	**– PreAMP**	**FF**	24.10	25.18	24.02	18.45	25.44	22.64	1.46	2.55	0.363	0.171
**Replica B**	**Kelly**	**– PreAMP**	**FF**	24.22	25.22	23.94	18.48	25.33	22.59	1.63	2.63	0.323	0.161
**Replica C**	**Kelly**	**– PreAMP**	**FF**	25.84	26.93	25.08	19.33	26.77	23.73	2.12	3.21	0.230	0.108
**Replica A**	**NCI-H2228**	**– PreAMP**	**FF**	40.00	26.31	25.32	18.00	24.29	22.54	17.46	3.77	0.000	0.073
**Replica B**	**NCI-H2228**	**– PreAMP**	**FF**	40.00	26.60	25.31	18.05	24.33	22.57	17.43	4.04	0.000	0.061
**Replica C**	**NCI-H2228**	**– PreAMP**	**FF**	38.09	28.17	26.34	18.81	25.59	23.58	14.51	4.59	0.000	0.041
**Replica A**	**A549**	**– PreAMP**	**FF**	40.00	37.16	25.45	18.04	26.23	23.24	16.76	13.92	0.000	0.000
**Replica B**	**A549**	**– PreAMP**	**FF**	40.00	38.30	25.60	19.27	26.37	23.75	16.26	14.56	0.000	0.000
**Replica C**	**A549**	**– PreAMP**	**FF**	40.00	39.19	26.54	19.38	27.19	24.37	15.63	14.82	0.000	0.000
**Replica A**	**Kelly**	**– PreAMP**	**FFPE**	29.70	29.38	28.27	21.59	29.11	26.33	3.37	3.06	0.097	0.120
**Replica B**	**Kelly**	**– PreAMP**	**FFPE**	29.70	29.37	28.35	21.33	29.09	26.26	3.44	3.11	0.092	0.116
**Replica C**	**Kelly**	**– PreAMP**	**FFPE**	30.06	29.67	28.44	21.57	29.20	26.40	3.66	3.27	0.079	0.103
**Replica A**	**NCI-H2228**	**– PreAMP**	**FFPE**	40.00	28.96	27.65	19.21	26.93	24.59	15.41	4.37	0.000	0.048
**Replica B**	**NCI-H2228**	**– PreAMP**	**FFPE**	37.07	28.96	27.55	19.24	26.79	24.52	12.55	4.43	0.000	0.046
**Replica C**	**NCI-H2228**	**– PreAMP**	**FFPE**	40.00	29.51	28.15	19.59	27.17	24.97	15.03	4.54	0.000	0.043
**Replica A**	**A549**	**– PreAMP**	**FFPE**	38.65	38.43	27.47	19.45	28.44	25.12	13.53	13.31	0.000	0.000
**Replica B**	**A549**	**– PreAMP**	**FFPE**	39.10	38.62	27.11	19.12	28.34	24.86	14.24	13.76	0.000	0.000
**Replica C**	**A549**	**– PreAMP**	**FFPE**	40.00	37.64	28.16	20.26	29.05	25.82	14.18	11.81	0.000	0.000
**Replica A**	**Kelly**	**+ PreAMP**	**FFPE**	21.99	21.70	20.36	13.59	21.68	18.55	3.44	3.15	0.092	0.113
**Replica B**	**Kelly**	**+ PreAMP**	**FFPE**	22.04	21.85	20.61	13.81	21.50	18.64	3.40	3.21	0.095	0.108
**Replica C**	**Kelly**	**+ PreAMP**	**FFPE**	21.65	21.42	20.11	13.48	21.30	18.30	3.35	3.12	0.098	0.115
**Replica A**	**NCI-H2228**	**+ PreAMP**	**FFPE**	31.34	21.44	19.79	11.43	19.17	16.79	14.55	4.64	0.000	0.040
**Replica B**	**NCI-H2228**	**+ PreAMP**	**FFPE**	32.96	21.85	20.14	11.76	19.34	17.08	15.88	4.77	0.000	0.037
**Replica C**	**NCI-H2228**	**+ PreAMP**	**FFPE**	32.90	21.07	19.38	11.07	18.67	16.37	16.52	4.70	0.000	0.039
**Replica A**	**A549**	**+ PreAMP**	**FFPE**	40.00	32.37	19.68	11.80	20.72	17.40	22.60	14.97	0.000	0.000
**Replica B**	**A549**	**+ PreAMP**	**FFPE**	40.00	31.69	21.02	12.87	21.39	18.43	21.57	13.26	0.000	0.000
**Replica C**	**A549**	**+ PreAMP**	**FFPE**	33.34	30.51	19.84	11.93	20.79	17.52	15.82	12.99	0.000	0.000

Mean Cq values of technical triplicates of the two ALK assays (5′ *ALK* and 3′ *ALK*) and three reference genes (*GUSB/GAPDH/TBP*) from three independent experiments (replica A, B and C) are reported. Assays were performed on fresh frozen (FF) and formalin-fixed, paraffin-embedded (FFPE) Kelly, NCI-H2228 and A549 cell samples in the absence (– PreAMP) or presence (+ PreAMP) of pre-amplification prior to PCR. Mean Cq values of the three reference genes, expression values of 5′ ($\Delta Cq_{5'ALK}$) and 3′ ($\Delta Cq_{3'ALK}$) ALK relative to the mean of the reference genes ($Cq_{5'ALK} - Cq_{MeanREFs}$ and $Cq_{3'ALK} - Cq_{MeanREFs}$), and relative expression levels of the 5′ ($2^{-\Delta Cq5'ALK}$) and 3′ ($2^{-\Delta Cq3'ALK}$) portions of the *ALK* transcript calculated using the formula $2^{-\Delta Cq}$ (where $\Delta Cq = Cq_{ALK}$ – average Cq_{REF}) are indicated.

Supplementary Table S2: Pre-amplification uniformity values relative to the two ALK assays measured in FFPE Kelly cells

		ΔΔCq ($\Delta Cq_{PreAmp} - \Delta Cq_{cDNA}$)		Mean Uniformity		STDEV Uniformity	
		5′ ALK	3′ ALK	5′ ALK	3′ ALK	5′ ALK	3′ ALK
+ PreAMP vs – PreAMP	FFPE Kelly A	0.07	0.09	– 0.09	0.01	0.20	0.14
+ PreAMP vs – PreAMP	FFPE Kelly B	– 0.04	0.10				
+ PreAMP vs – PreAMP	FFPE Kelly C	– 0.31	–0.15				

Pre-amplification uniformity values ($\Delta\Delta Cq = \Delta Cq_{PreAmp} - \Delta Cq_{cDNA}$) calculated in three independent experiments (replica A, B and C) in Kelly cells positive both for the 5′ and the 3′ portion of *ALK*. For each of the two *ALK* assays (5′ *ALK* and 3′ *ALK*), mean uniformity values (Mean Uniformity), and relative standard deviations (STDEV Uniformity) are indicated.

Supplementary Table S3: Improvements to Cq values in pre-amplified FFPE Kelly, NCI-H2228 and A549 cells

		5′ ALK		*3′ ALK*		*GUSB*		*GAPDH*		*TBP*		Mean Cq Improvements	STDEV Mean Cq Improvements
		Mean Cq	ΔCq	Mean Cq	ΔCq	Mean Cq	ΔCq	Mean Cq	ΔCq	Mean Cq	ΔCq		
FFPE Kelly	– PreAMP	29.82	– 7.93	29.47	– 7.82	28.35	– 7.99	21.50	– 7.87	29.13	– 7.64	– 7.85	0.13
	+ PreAMP	21.89		21.66		20.36		13.63		21.50			
FFPE NCI-H2228	– PreAMP	39.02	NA	29.14	– 7.69	27.78	– 8.01	19.34	– 7.92	26.96	– 7.90	– 7.88	0.14
	+ PreAMP	32.40		21.45		19.77		11.42		19.06			
FFPE A549	– PreAMP	39.25	NA	38.23	NA	27.58	– 7.40	19.61	– 7.41	28.61	– 7.65	– 7.49	0.14
	+ PreAMP	37.78		31.52		20.18		12.20		20.97			

Difference (ΔCq) of mean Cq values (from three experiments) relative to the two ALK assays (5′ *ALK* and 3′ *ALK*) and the three reference genes (*GUSB/GAPDH/TBP*) in pre-amplified (+ PreAMP) *vs*. not pre-amplified (– PreAMP) FFPE Kelly, NCI-H2228 and A549 cells. Mean Cq improvements and associated standard deviations (STDEV Mean Cq improvements) are reported.

Supplementary Table S4: Comparison of Cq values in pre-amplified and not pre-amplified Kelly calibration curves

Dilution Series (%)	5′ *ALK*					3′ *ALK*				
	–PreAMP	+PreAMP	ΔCq (Cq-PreAMP – Cq + PreAMP)	Mean ΔCq	Stdev Mean Cq	–PreAMP	+PreAMP	ΔCq ($Cq_{-PreAMP}$ – $Cq_{+PreAMP}$)	Mean ΔCq	Stdev Mean ΔCq
**100**	29.41	21.34	8.07	7.57	0.40	29.00	21.34	7.66	7.38	0.35
**90**	29.40	21.73	7.67			28.92	21.67	7.25		
**80**	29.52	21.88	7.64			29.16	21.85	7.31		
**70**	29.69	22.08	7.61			29.37	22.05	7.31		
**60**	29.80	22.38	7.42			29.05	22.32	6.73		
**50**	30.11	22.63	7.48			29.88	22.50	7.38		
**45**	30.28	22.76	7.52			30.05	22.73	7.32		
**40**	30.57	22.77	7.80			30.19	22.77	7.42		
**35**	30.76	23.11	7.65			30.47	23.04	7.43		
**30**	32.10	23.78	8.31			31.69	23.63	8.07		
**25**	30.99	23.71	7.28			30.85	23.67	7.18		
**22.5**	31.24	24.20	7.04			30.99	24.00	6.99		
**20**	31.43	24.45	6.98			30.73	24.13	6.60		
**17.5**	32.01	24.28	7.72			31.73	24.17	7.56		
**15**	31.73	24.49	7.24			31.51	24.43	7.08		
**12.5**	31.99	24.90	7.09			31.89	24.93	6.96		
**10**	32.36	25.03	7.33			32.06	24.86	7.20		
**7.5**	33.03	25.27	7.76			32.73	25.16	7.57		
**5**	33.00	25.82	7.18			33.20	25.49	7.71		
**4**	34.43	25.95	8.49			33.52	25.82	7.70		
**3**	34.02	26.65	7.37			34.03	26.29	7.74		
**2**	35.03	26.95	8.08			34.55	26.85	7.70		
**1**	35.18	27.90	7.28			35.56	27.80	7.76		

Percentages (%) of the dilution series used to generate the calibration curves are indicated. The table also reports: i) the mean Cq values of technical triplicates measured without (– PreAMP) and with (+ PreAMP) pre-amplification; ii) the difference between the Cq values with and without pre-amplification for each data point of the curve (ΔCq = Cq-PreAMP – Cq + PreAMP); iii) the mean difference in Cq across all the data points (Mean ΔCq) of the curve and the relative standard deviation (Stdev Mean ΔCq) for both the 5′ and 3′ ALK assays.

Supplementary Table S5: Parameters of the different models generated with external calibration curves

		3′ ALK				*5′ ALK*			
		**NCI-H2228**	**NCI-H2228**	**Kelly**	**Kelly**	**Kelly + NCI-H2228**	**Kelly + NCI-H2228**	**Kelly**	**Kelly**
		**Dilution range: 1%–100%**	**Dilution range: 1%–50%**	**Dilution range: 1%–100%**	**Dilution range: 1%–50%**	**Dilution range: 1%–100%**	**Dilution range: 1%–50%**	**Dilution range: 1%–100%**	**Dilution range: 1%–50%**
A) Linear regression model dependent variable: normalized Cq predictor: percentage (log-transformed, base 10) of total ALK positive RNA									
**Number of observations**	**N**	23	18	23	18	46	36	23	18
**Intercept**	**a**	10.14710	10.23355	11.02475	10.79951	10.58592	10.51653	11.24635	11.0053
**Regression coefficient**	**Byx**	– 2.81736	– 2.92289	– 3.56424	– 3.28623	– 3.1908	– 3.10456	– 3.65178	– 3.3542
**Standard error of regression coefficient**	**Sb**	0.04972	0.05387	0.09279	0.06808	0.07675	0.06071	0.10123	0.07914
**Mean square error**	**S2yx**	0.01679	0.01212	0.05849	0.01935	0.08004	0.03079	0.06961	0.02616
**Dependent mean**	**Ybar**	6.59754	7.06759	6.5342	7.24000	6.56587	7.1538	6.64551	7.37222
B) Linear regression model dependent variable: percentage (log-transformed, base 10) of total ALK positive RNA predictor: normalized Cq									
**Dependent mean**	**Xbar**	1.25989	1.08316	1.25989	1.08316	1.25989	1.08316	1.25989	1.08316
**Total sum of squares**	**Sxx**	6.79279	4.17592	6.79279	4.17592	13.58557	8.35184	6.79279	4.17592

Parameters relative to the various algorithms we derived for inverse prediction are reported. A) Linear regression model with normalized Cq as dependent variable and percentage (log-transformed, base 10) of total *ALK* positive RNA as predictor. B) Linear regression model with percentage (log-transformed, base 10) of total *ALK* positive RNA as dependent variable and normalized Cq as predictor. Calibration curves generated using 100 ng of input RNA.

Supplementary Table S6A: Comparison of the different prediction models in 20 FFPE lung non-pathological samples

Case ID	3′ *ALK* NCI-H2228 1–100%	3′ *ALK* NCI-H2228 1–50%	3′ *ALK* KELLY 1–100%	3′ *ALK* KELLY 1–50%	3′ *ALK* NCI-H2228+KELLY 1–100%	3′ *ALK* NCI-H2228+KELLY 1–50%	5′ *ALK* KELLY 1–100%	5′ *ALK* KELLY 1–50%
**1-NP**	6.6 (5.2–8.3)	6.6 (5.4–8.0)	7.8 (5.6–10.9)	8.0 (6.4–9.8)	7.3 (4.8–11.0)	7.3 (5.6–9.5)	0	0
**2-NP**	7.8 (6.2–9.8)	7.8 (6.4–9.4)	9.0 (6.4–12.5)	9.2 (7.4–11.4)	8.4 (5.5–12.8)	8.5 (6.5–11.1)	3.9 (2.7–5.6)	3.7 (2.9–4.8)
**3-NP**	0	0	0	0	0	0	0	0
**4-NP**	1.0 (0.8–1.3)	1.1 (0.9–1.3)	1.8 (1.2–2.5)	1.6 (1.2–2.0)	1.4 (0.9–2.1)	1.3 (1.0–1.7)	0	0
**5-NP**	6.2 (4.9–7.8)	6.2 (5.1–7.5)	7.5 (5.3–10.4)	7.6 (6.1–9.4)	6.9 (4.5–10.5)	6.9 (5.3–9.0)	1.3 (0.9–1.8)	1.1 (0.8–1.4)
**6-NP**	1.0 (0.8–1.3)	1.1 (0.9–1.3)	1.7 (1.2–2.5)	1.6 (1.2–2.0)	1.4 (0.9–2.1)	1.3 (1.0–1.7)	0	0
**7-NP**	1.5 (1.2–2.0)	1.6 (1.3–2.0)	2.5 (1.7–3.5)	2.3 (1.8–2.9)	2.0 (1.3–3.1)	1.9 (1.5–2.6)	1.9 (1.3–2.7)	1.7 (1.3–2.2)
**8-NP**	8.0 (6.3–10.0)	7.9 (6.5–9.5)	9.1 (6.5–12.7)	9.3 (7.5–11.6)	8.6 (5.6–13.0)	8.6 (6.6–11.3)	0	0
**9-NP**	1.2 (0.9–1.5)	1.3 (1.0–1.6)	2.0 (1.4–2.9)	1.8 (1.5–2.3)	1.6 (1.0–2.5)	1.5 (1.2–2.0)	0	0
**10-NP**	9.1 (7.3–11.5)	9.0 (7.5–10.9)	10.1 (7.2–14.1)	10.5 (8.5–13)	9.7 (6.4–14.7)	9.8 (7.5–12.8)	0	0
**11-NP**	10.2 (8.1–12.7)	10.0 (8.3–12.1)	11.0 (7.9–15.4)	11.5 (9.3–14.3)	10.6 (7.0–16.1)	10.8 (8.2–14.1)	0	0
**12-NP**	0.7 (0.6–0.9)	0.8 (0.6–1.0)	1.4 (0.9–1.9)	1.2 (0.9–1.5)	1.0 (0.7–1.6)	1.0 (0.7–1.3)	1.4 (0.9–2.0)	1.2 (0.9–1.6)
**13-NP**	2.4 (1.9–3.0)	2.5 (2.0–3.0)	3.5 (2.5–5.0)	3.4 (2.7–4.2)	3.0 (1.9–4.6)	2.9 (2.2–3.8)	0	0
**14-NP**	1.2 (0.9–1.5)	1.2 (1.0–1.5)	2.0 (1.4–2.8)	1.8 (1.4–2.3)	1.6 (1.0–2.4)	1.5 (1.1–2.0)	1.6 (1.1–2.3)	1.4 (1.1–1.8)
**15-NP**	2.7 (2.1–3.4)	2.7 (2.3–3.3)	3.8 (2.7–5.4)	3.7 (2.9–4.5)	3.3 (2.1–5.0)	3.2 (2.4–4.2)	1.1 (0.7–1.6)	1.0 (0.7–1.2)
**16-NP**	0.7 (0.5–0.9)	0.7 (0.6–0.9)	1.3 (0.9–1.9)	1.1 (0.9–1.4)	1.0 (0.6–1.5)	0.9 (0.7–1.2)	0	0
**17-NP**	0.3 (0.2–0.4)	0.3 (0.2–0.4)	0.6 (0.4–0.9)	0.5 (0.4–0.7)	0.4 (0.3–0.7)	0.4 (0.3–0.6)	0.9 (0.6–1.3)	0.7 (0.6–1.0)
**18-NP**	2.6 (2.0–3.2)	2.6 (2.2–3.2)	3.7 (2.6–5.2)	3.5 (2.8–4.4)	3.1 (2.0–4.8)	3.1 (2.3–4.0)	0	0
**19-NP**	0	0	0	0	0	0	0	0
**20–NP**	2.4 (1.9–3.0)	2.5 (2.0–3.0)	3.5 (2.5–5.0)	3.4 (2.7–4.2)	3.0 (1.9–4.6)	2.9 (2.2–3.8)	0	0

Sample number (Case ID) and percentage of prediction with associated confidence intervals for the 3′ and the 5′ portion of *ALK* in each of the indicated models in non`pathological (NP) samples ($N = 20$) are reported.

Supplementary Table S6B: Normal sample distribution

Calibration Curve	*N*	Median	Lower quartile	Upper quartile	95th percentile	Min	Max
**3′ ALK NCI-H2228 1–100%**	20	2.0	0.9	6.4	9.7	0.0	10.2
**3′ ALK NCI-H2228 1–50%**	20	2.1	1.1	6.4	9.5	0.0	10.0
**3′ ALK KELLY 1–100%**	20	3.0	1.6	7.7	10.6	0.0	11.0
**3′ ALK KELLY 1–50%**	20	2.9	1.4	7.8	11.0	0.0	11.5
**3′ ALK NCI-H2228 + KELLY 1–100%**	20	2.5	1.2	7.1	10.2	0.0	10.6
**3′ ALK NCI-H2228+KELLY 1–50%**	20	2.4	1.2	7.1	10.3	0.0	10.8
**5′ ALK KELLY 1–100%**	20	0.0	0.0	1.2	2.9	0.0	3.9
**5′ ALK KELLY 1–50%**	20	0.0	0.0	1.1	2.7	0.0	3.7

The median, lower quartile, upper quartile, and 95th percentile of the predicted positivity (with minimum, Min and maximum, Max of predicted percentage) for each of the six models for the 3′ and the two models for the 5′ portion of *ALK* are also shown.

Supplementary Table S7: Distribution of the Cq values relative to the three reference genes in the 51 FFPE NSCLC samples

	N	Median	Q1	Q3	Min	Max	IQR	Outlier Range	
GUSB	51	21.25	19.96	22.45	18.53	24.96	2.49	16.22	26.19
TBP	51	23.37	22.23	24.46	20.99	27.47	2.23	18.89	27.81
GAPDH	51	15.60	14.54	16.91	12.80	18.85	2.37	10.98	20.47
**Mean REFs**	51	19.91	18.70	21.24	17.73	23.57	2.54	14.88	25.06

Median values, lower quartile (Q1), upper quartile (Q3), minimum (Min), maximum (Max), interquartile range (IQR), and the outlier range for each of the three reference genes (*GUSB*, *TBP*, and *GAPDH*), singly or averaged (Mean REFs), in the 51 FFPE NSCLC samples are reported.

Supplementary Table S8: Comparison of the different prediction models in the 51 FFPE NSCLC samples

CASE ID	FISH ALK STATUS	3′ *ALK* NCI-H2228 1–100%	3′ *ALK* NCI-H2228 1–50%	3′ *ALK* KELLY 1–100%	3′ *ALK* KELLY 1–50%	3′ *ALK* NCI-H2228+KELLY 1–100%	3′ *ALK* NCI-H2228+KELLY 1–50%	5′ *ALK* KELLY 1–100%	5′ *ALK* KELLY 1–50%
**1**	POS	100 (80.1–100)	91.5 (74.8–100)	67.7 (48.4–95.5)	82.6 (66.0–100)	80.8 (53.1–100)	86.7 (65.8–100)	0	0
**2**	POS	100 (100–100)	100 (100–100)	100 (89.3–100)	100 (100–100)	100 (100–100)	100 (100–100)	0	0
**3**	POS	100 (100–100)	100 (100–100)	100 (100–100)	100 (100–100)	100 (100–100)	100 (100–100)	0	0
**4**	POS	100 (100–100)	100 (94.4–100)	82.2 (58.6–100)	100 (81.1–100)	100 (65.9–100)	100 (82.0–100)	0	0
**5**	POS	100 (100–100)	100 (100–100)	100 (100–100)	100 (100–100)	100 (100–100)	100 (100–100)	0	0
**6**	POS	100 (100–100)	100 (100–100)	98.5 (70.1–100)	100 (98.4–100)	100 (80.5–100)	100 (100–100)	0	0
**7**	POS	100 (100–100)	100 (100–100)	100 (100–100)	100 (100–100)	100 (100–100)	100 (100–100)	0	0
**8**	POS	100 (100–100)	100 (100–100)	100 (100–100)	100 (100–100)	100 (100–100)	100 (100–100)	0	0
**9**	POS	100 (100–100)	100 (100–100)	100 (100–100)	100 (100–100)	100 (100–100)	100 (100–100)	0	0
**10**	POS	100 (100–100)	100 (100–100)	100 (100–100)	100 (100–100)	100 (100–100)	100 (100–100)	0	0
**11**	POS	90.8 (72.1–100)	82.6 (67.6–100)	62.3 (44.5–87.7)	75.4 (60.3–95.0)	73.6 (48.4–100)	78.7 (59.8–100)	0	0
**12**	POS	100 (100–100)	100 (100–100)	100 (100–100)	100 (100–100)	100 (100–100)	100 (100–100)	0	0
**13**	POS	100 (97.3–100)	100 (90.1–100)	79.1 (56.4–100)	97.7 (77.8–100)	96.1 (63.1–100)	100 (78.4–100)	0	0
**14**	POS	100 (89.8–100)	100 (83.4–100)	74.1 (52.9–100)	91.1 (72.6–100)	89.4 (58.7–100)	96.2 (72.9–100)	0	0
**15**	POS	63.9 (50.9–80.6)	58.9 (48.4–72.0)	47.2 (33.8–66.2)	55.8 (44.8–69.9)	53.9 (35.5–82.3)	57.2 (43.6–75.6)	8.5 (5.9–12.1)	8.7 (6.8–11.0)
**16**	POS	100 (100–100)	100 (100–100)	100 (100–100)	100 (100–100)	100 (100–100)	100 (100–100)	0	0
**17**	POS	100 (100–100)	100 (100–100)	100 (100–100)	100 (100–100)	100 (100–100)	100 (100–100)	0	0
**18**	NEG	0	0	0	0	0	0	0	0
**19**	NEG	67.7 (53.9–85.4)	62.2 (51.1–76.2)	49.3 (35.3–69.3)	58.6 (47.0–73.5)	56.7 (37.4–86.6)	60.3 (45.9–79.6)	0	0
**20**	NEG	12.2 (9.7–15.2)	11.9 (9.8–14.4)	12.7 (9.1–17.7)	13.5 (10.9–16.6)	12.5 (8.2–18.9)	12.7 (9.7–16.6)	10.5 (7.4–15.0)	11 (8.6–14.0)
**21**	NEG	3.5 (2.8–4.4)	3.6 (3.0–4.4)	4.8 (3.4–6.7)	4.6 (3.7–5.7)	4.2 (2.7–6.3)	4.1 (3.1–5.4)	1.4 (0.9–2.0)	1.2 (0.9–1.5)
**22**	NEG	5.0 (3.9–6.2)	5.0 (4.1–6.1)	6.2 (4.4–8.7)	6.2 (5.0–7.7)	5.6 (3.7–8.6)	5.6 (4.3–7.3)	0	0
**23**	NEG	2.8 (2.2–3.6)	2.9 (2.4–3.5)	4.0 (2.8–5.6)	3.8 (3.1–4.8)	3.4 (2.2–5.2)	3.4 (2.6–4.4)	0	0
**24**	NEG	0.5 (0.4–0.6)	0.5 (0.4–0.6)	1.0 (0.7–1.4)	0.8 (0.6–1.0)	0.7 (0.4–1.1)	0.7 (0.5–0.9)	0	0
**25**	NEG	14.3 (11.4–17.9)	13.9 (11.5–16.8)	14.5 (10.4–20.1)	15.5 (12.5–19.2)	14.4 (9.5–21.8)	14.7 (11.3–19.3)	21.9 (15.4–31.3)	24.4 (19.2–31.3)
**26**	NEG	4.3 (3.4–5.4)	4.3 (3.6–5.3)	5.6 (3.9–7.8)	5.5 (4.4–6.8)	4.9 (3.2–7.5)	4.9 (3.7–6.4)	0	0
**27**	NEG	3.3 (2.6–4.2)	3.4 (2.8–4.2)	4.6 (3.2–6.4)	4.4 (3.6–5.5)	4.0 (2.6–6.1)	3.9 (3.0–5.2)	5.0 (3.5–7.2)	4.9 (3.8–6.3)
**28**	NEG	2.4 (1.9–3.0)	2.5 (2.0–3.0)	3.5 (2.5–4.9)	3.3 (2.7–4.1)	3.0 (1.9–4.5)	2.9 (2.2–3.8)	0	0

**29**	NEG	1.2 (0.9–1.5)	1.2 (1.0–1.5)	2.0 (1.4–2.8)	1.8 (1.4–2.2)	1.6 (1.0–2.4)	1.5 (1.1–2.0)	0	0
**30**	NEG	0	0	0	0	0	0	0	0
**31**	NEG	1.3 (1.0–1.7)	1.4 (1.1–1.7)	2.2 (1.5–3.1)	2.0 (1.6–2.5)	1.7 (1.1–2.7)	1.7 (1.3–2.2)	0	0
**32**	NEG	4.2 (3.3–5.3)	4.3 (3.5–5.2)	5.5 (3.9–7.7)	5.4 (4.4–6.7)	4.9 (3.2–7.4)	4.8 (3.7–6.3)	2.2 (1.5–3.1)	2.0 (1.5–2.5)
**33**	NEG	0	0	0	0	0	0	0	0
**34**	NEG	7.4 (5.9–9.3)	7.4 (6.1–9.0)	8.6 (6.2–12.0)	8.8 (7.1–10.9)	8.1 (5.3–12.3)	8.1 (6.2–10.6)	12.3 (8.6–17.6)	13.1 (10.3–16.7)
**35**	NEG	0	0	0	0	0	0	0	0
**36**	NEG	0	0	0	0	0	0	0	0
**37**	NEG	0	0	0	0	0	0	0	0
**38**	NEG	0	0	0	0	0	0	0	0
**39**	NEG	3.2 (2.5–4.0)	3.3 (2.7–4.0)	4.4 (3.1–6.2)	4.3 (3.4–5.3)	3.8 (2.5–5.8)	3.8 (2.9–5.0)	2.7 (1.8–3.8)	2.4 (1.9–3.1)
**40**	NEG	2.0 (1.6–2.6)	2.1 (1.7–2.6)	3.1 (2.2–4.3)	2.9 (2.3–3.6)	2.6 (1.7–3.9)	2.5 (1.9–3.3)	0	0
**41**	NEG	1.1 (0.9–1.4)	1.2 (0.9–1.4)	1.9 (1.3–2.7)	1.7 (1.4–2.1)	1.5 (1.0–2.3)	1.4 (1.1–1.9)	0	0
**42**	NEG	0	0	0	0	0	0	0	0
**43**	NEG	0	0	0	0	0	0	0	0
**44**	NEG	0.5 (0.4–0.6)	0.5 (0.4–0.7)	1.0 (0.7–1.4)	0.9 (0.7–1.1)	0.7 (0.5–1.1)	0.7 (0.5–0.9)	0	0
**45**	NEG	0	0	0	0	0	0	0	0
**46**	NEG	2.6 (2.0–3.3)	2.7 (2.2–3.3)	3.7 (2.6–5.3)	3.6 (2.9–4.4)	3.2 (2.1–4.9)	3.1 (2.4–4.1)	6.5 (4.5–9.3)	6.5 (5.1–8.3)
**47**	NEG	0.5 (0.4–0.7)	0.6 (0.5–0.7)	1.1 (0.7–1.5)	0.9 (0.7–1.1)	0.8 (0.5–1.2)	0.7 (0.5–1.0)	0	0
**48**	NEG	4.8 (3.8–6.0)	4.8 (4.0–5.8)	6.0 (4.3–8.4)	6.0 (4.8–7.4)	5.4 (3.6–8.3)	5.4 (4.1–7.1)	0	0
**49**	NEG	1.2 (0.9–1.5)	1.2 (1.0–1.5)	2.0 (1.4–2.8)	1.8 (1.4–2.2)	1.6 (1.0–2.4)	1.5 (1.1–2.0)	0	0
**50**	NEG	0	0	0	0	0	0	0	0
**51**	NEG	0	0	0	0	0	0	0	0
**FFPE Kelly**	–	100 (100–100)	100 (100–100)	100 (100–100)	100 (100–100)	100 (100–100)	100 (100–100)	100 (100–100)	100 (100–100)
**FFPE NCI-H2228**	–	85.8 (68.1–100)	78.2 (64.0–96.0)	59.5 (42.6–83.8)	71.8 (57.4–90.3)	69.9 (46.0–100)	74.7 (56.8–99.0)	0	0
**FFPE A549**	–	0	0	0	0	0	0	0	0

Sample number (Case ID) and percentage of prediction with associated 95% confidence intervals for the 3′ and the 5′ portion of *ALK* in each of the indicated models are reported. Binary *ALK* status by FISH analysis is indicated as positive (POS, ≥ 15% of positive nuclei) or negative (NEG, < 15% of positive nuclei).

Supplementary Table S9: Design details for each TaqMan gene expression assay used in PCR analysis

Gene Symbol	Assay ID	Ref Seq	Translated Protein	Exon Boundary	Assay Location	Amplicon Length	Amplicon Context Sequence
**ALK**	Hs00608292_m1	NM_004304.4	NP_004295.2	27–28	5022	59	AAAAGCAACCAGGAA<br>GTTCTGGAGTTTGTCA<br>CCAGTGGAGGCCGGA<br>TGGACCCACCCAAGA<br>ACTGCCCTGGGCCTGT<br>ATACCGGATAATGACT<br>CAGTGCTGGCAACATC<br>AGCCTGAAGACAGGC<br>CCAACTTTGCCATCAT<br>TTTGG
**ALK**	Hs01058323_m1	NM_004304.4	NP_004295.2	9–10	2775	66	GGTCTGGCATGTCGCC<br>GCCTATGAAGGCTTGA<br>GCCTGTGGCAGTGGAT<br>GGTGTTGCCTCTCCTC<br>GATGTGTCTGACAGGT<br>TCTGGCTGCAGATGGT<br>CGCATGGTGGGGACAA<br>GGATCCAGAGCCATCG<br>TGGCTTTTGACAATATC<br>TCCATCAGCCTGGACTG
**GAPDH**	Hs_03929097_g1	NM_001256799.1<br>NM_002046.4	NP_001243728.1<br>NP_002037.2	8–8<br>9–9	1250<br>1226	58	CATGGCCCACATGGCC<br>TCCAAGGAGTAAGACC<br>CCTGGACCACCAGCCC<br>CAGCAAGAGCACAAG<br>AGGAAGAGAGAGACC<br>CTCACTGCTGGGGAGT<br>CCCTGCCACACTCAGT<br>CCCCCACCACACTGAA<br>TCTCCCCTCCTCACAGT
**GUSB**	Hs99999908_m1	NM_000181.3	NP_000172.2	11–12	1925	81	GCAGTACCATCTGGGTC<br>TGGATCAAAAACGCAG<br>AAAATACGTGGTTGGA<br>GAGCTCATTTGGAATT<br>TTGCCGATTTCATGACT<br>GAACAGTCACCGACGA<br>GAGTGCTGGGGAATAA<br>AAAGGGGATCTTCAC<br>TCGGCAGAGACAACCA<br>AAAAGTGCAGCGTTCC<br>TTTTGCGAGAGAGATA<br>CTGGAAGATTGC
**TBP**	Hs00427621_m1	NM_001172085.1<br>NM_003194.4	NP_001165556.1<br>NP_003185.1	3–4<br>4–5	666<br>868	65	CAGTGAATCTTGGTTG<br>TAAACTTGACCTAAAG<br>ACCATTGCACTTCGTG<br>CCCGAAACGCCGAAT<br>ATAATCCCAAGCGGTT<br>TGCTGCGGTAATCATG<br>AGGATAAGAGAGCCA<br>CGAACCACGGCACTG<br>ATTTTCAGTTCTGG<br>GAAAATGGTGTGCAC<br>AGG

Gene name (Gene Symbol), Identification number (Assay ID) of each TaqMan assay, accession number of the transcripts (RefSeq) or the corresponding translated protein recognized by the assay, exon boundary, assay location, amplicon length and estimated maximal amplicon context sequence (measured by adding the length of the amplicon to either side of the approximate probe sequence highlighted in blue) are reported. The nucleotide base located in the center of the probe, corresponding to assay location, is highlighted in yellow; the approximate location of the probe (measured by including 13 bases on either side of the assay location given that probes are typically 18–25 nucleotides in length) is highlighted in blue.
